# Protein Nanoparticles: Uniting the Power of Proteins with Engineering Design Approaches

**DOI:** 10.1002/advs.202104012

**Published:** 2022-01-25

**Authors:** Nahal Habibi, Ava Mauser, Yeongun Ko, Joerg Lahann

**Affiliations:** ^1^ Biointerfaces Institute Department of Chemical Engineering University of Michigan Ann Arbor MI 48109 USA; ^2^ Biointerfaces Institute Department of Biomedical Engineering University of Michigan Ann Arbor MI 48109 USA; ^3^ Biointerfaces Institute Departments of Chemical Engineering Material Science and Engineering Biomedical Engineering and Macromolecular Science and Engineering University of Michigan Ann Arbor MI 48109 USA

**Keywords:** biomimetic, electrospraying, nanomedicine, protein nanoparticle, self‐assembly

## Abstract

Protein nanoparticles, PNPs, have played a long‐standing role in food and industrial applications. More recently, their potential in nanomedicine has been more widely pursued. This review summarizes recent trends related to the preparation, application, and chemical construction of nanoparticles that use proteins as major building blocks. A particular focus has been given to emerging trends related to applications in nanomedicine, an area of research where PNPs are poised for major breakthroughs as drug delivery carriers, particle‐based therapeutics or for non‐viral gene therapy.

## Graphical abstract

Protein nanoparticles (PNPs) have recently gained significant momentum. Advances in protein engineering combined with precise engineering approaches are poised to propel protein‐based delivery systems to the forefront of nanomedicine. This review summarizes emerging technologies, potential opportunities and current challenges of PNPs.

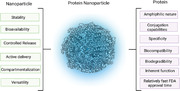

## 1 Introduction

Proteins are central to biological function.^[^ ^1^, ^2^ ^]^ They are macromolecular molecules involved in a myriad of biological functions ranging from DNA repair, catalysis of metabolic reactions to cell signaling. To fully appreciate the specificity and efficiency with which proteins operate, their hierarchal structure and their interactions in biological environments must be considered. The primary structure, that is, the sequence with which the 20 natural amino acids are lined up via peptide bonds, encodes a unique bar code that characteristically identifies each protein.^[^ ^3^ ^]^ The secondary structure relates to conformational motifs such as alpha helices, beta sheets, and conformational turns of the amino acid chains. A tertiary structure of the protein emerges from longer‐range intramolecular interactions involving disulfide bridges, hydrogen bonding, or Van der Waals, hydrophobic, and electrostatic interactions, whereas its quaternary structure further considers intermolecular interactions. To emphasize how impactful even minute changes in a protein's composition can be, consider sickle cell anemia.^[^ ^4^ ^]^ Hemoglobin, the protein contained in red blood cells, has a quaternary structure that includes two alpha and two beta chains. A single base substitution of the more apolar valine instead of glutamic acid promotes hydrophobic interactions with an adjacent beta chain and ultimately alters the shape of the cell.^[^ ^4^ ^]^ This change in structure increases the rigidity of the membrane and blocks effective transport through capillaries of the body.

In the last 20 years, the number of known protein structures have increased by ten times with over 170 000 structures available in 2021 according to the Protein Data Bank.^[^ ^5^ ^]^ The increased rate with which these proteins are discovered resulted in an improved mechanistic understanding of how proteins interact with each other, and their biological environments. A particular hotspot of contemporary research is the development of protein therapeutics.^[^ ^1^, ^2^ ^]^ As therapeutical agents, proteins leverage several innate properties such as specificity, environmental tolerance, recombinant production,^[^ ^6^ ^]^ and, at least in the case of human proteins, may benefit from faster regulatory approval processes.^[^ ^1^ ^]^ Not only protein conjugates, monoclonal antibodies, or enzymes, but also protein nanoparticles (PNPs) feature these advantageous characteristics.^[^ ^7^ ^]^ PNPs can exhibit characteristics from both proteins like inherent function, specificity, high degree of modification flexibility while leveraging the advantages of nanoparticles: controlled release, improved bioavailability, and stability. While PNPs have not been as extensively researched compared to other nanoparticle platforms, the idea of using proteins as cargo carriers is not new. The epitome of an ideal nanoparticle is a natural virus; viruses are structurally ordered, possess precise surface topologies, and can release cargo through controlled mechanisms, all of which was established through evolution.^[^ ^8^, ^9^ ^]^ Consequently, this has enabled viruses to effectively negotiate biological barriers, achieve high levels of specificity for targeted host cells, and even evade immunological responses. Using viruses as inspiration for drug delivery vehicles, engineered viral vectors like lentiviruses, adenoviruses and adeno‐associated viruses have found applications in gene therapy ranging from cancer to infectious and inflammatory diseases.^[^ ^10^ ^]^ Furthermore, drawing on inspiration from viral vectors, virus‐like particles (VLPs) have emerged as another type of protein nanoparticle. They are devoid of all genetic material, and are self‐assembled through viral proteins and represent a safer version of the viral vectors.^[^ ^11^ ^]^ Outside of viral vectors and VLPs, protein nanoparticles can take a third, more modular form: engineered protein nanoparticles. Engineered PNPs have advantages over viral vectors such as lower immunological responses and may address concerns of production when compared to VLPs. These engineered PNPs can be produced from a various sources of proteins (from plant to serum) and has been exploited in various applications ranging from biotechnology to food industries (**Table** **1**).^[^ ^12^, ^13^, ^14^, ^15^, ^16^, ^17^, ^18^, ^19^ ^]^

**Table 1:** Various applications of PNPs

Material/platform	Applications	Ref.
Human serum albumin (HSA)	Targeted drug and gene delivery	[20, 21, 22]
Keratin	Mucosal drug delivery	[23]
Virus‐like particles and caged protein	Cancer vaccine development	[24]
Antifreeze protein‐containing T33‐21 multimers	Freezing point depression enhancement, cryobiology	[25]
Rice bran oil‐based soy	Fortification of non‐dairy food products	[26]
Cereal‐based proteins (maize zein, wheat gliadin)	Food dispersions stabilizer	[27]
Bovine serum albumin (BSA)	Targeted drug delivery, pH sensor	[28, 29, 30, 31]
Z‐Elastin‐like polypeptide‐E2	Affinity precipitation of antibodies	[32]

The prospects of PNPs for a range of applications as listed in Table 1 hinge upon a range of tangible advantages including versatility, conjugation capabilities, biodegradability, availability, and affordability and relatively low immunogenicity.

i) Versatility: The range of available proteins that can be used for PNPs is vast, providing unprecedented versatility of PNPs, yet, the design space goes beyond natural proteins, as recombinant protein technologies have been effectively used. The design space for proteins is almost unlimited. For a 200‐residue protein, 20 ^200^ possible amino‐acid sequences exist, yet only a small subset has been sampled by evolutionary processes.^[^ ^33^ ^]^
ii) Conjugation capabilities: The type of amino acids and their side chain within the primary structure of proteins offer a variety of binding sites that can be exploited for subsequent conjugation or surface modification of PNPs.^[^ ^34^, ^35^ ^]^ Proteins have been used for covalent incorporation of dyes, hydrophobic drugs, or other active ingredients.^[^ ^13^, ^36^, ^37^, ^38^ ^]^ Apart from chemically conjugating to functional groups, the ligand‐binding properties of proteins can be utilized for surface modification. For example, it was shown that both BSA PNPs bound to immobilized anti‐BSA polyclonal antibodies and PNPs made from monoclonal antibodies against hepatitis B virus S antigen demonstrated specific binding to the corresponding antigen.^[^ ^39^ ^]^ The distinct binding sites of proteins are also widely exploited in antibody purification strategies.^[^ ^32^ ^]^ Specifically, PNPs were incorporated into affinity precipitation techniques, which combine the high selectivity of an affinity ligand with advantages of precipitation technologies.^[^ ^32^, ^40^ ^]^
iii) Biodegradability: Compared to conventional synthetic nanoparticles, like polymer‐based nanoparticles, another crucial property defining PNPs is the fact that their constituents can be cleaved by proteolytic enzymes. For example, Langer et al. studied the enzymatic degradation of human serum albumin (HSA) nanoparticles.^[^ ^41^ ^]^ Both intestinal and gastric enzymes effectively degraded HSA PNPs that were crosslinked with glutaraldehyde.^[^ ^41^ ^]^ The degradation kinetics were shown to be dependent on the degree of crosslinking with highly crosslinked PNPs requiring longer time for degradation. In addition to intestinal and gastric enzymes, the intracellular enzyme cathepsin B was also able to degrade crosslinked HSA PNPs, suggesting the degradability of PNPs after cell uptake.^[^ ^41^ ^]^
iv) Availability and affordability: PNPs based on soy proteins, for example, are abundant, low‐cost, and renewable, with increasing industrial interest in food applications.^[^ ^42^, ^43^, ^44^, ^45^ ^]^ Soy PNPs further contain protected polyunsaturated fatty acids. Similarly, rice bran oil‐based soy PNPs are used in soy yogurts to promote antiradical scavenging.^[^ ^26^ ^]^ In another food‐related application, PNPs are used as emulsifiers to stabilize food dispersions.^[^ ^27^ ^]^ As an example, cereal protein‐based nanoparticles, mainly made from wheat gliadin and maize zein proteins, have gained broader attention as stabilizers of air‐water or oil‐water interfaces.^[^ ^27^ ^]^
v) Immunogenicity: Proteins of human origin, such as HSA or lipoproteins are particularly well‐suited for applications where low immunogenicity is required.^[^ ^46^ ^]^ The immunogenicity of biopharmaceutical protein formulations is an important area of research and relies on combinations of multiple methods such as in silico‐modeling, cell culture tests, and animal studies.^[^ ^47^ ^]^ In addition to size, composition, and dose, critical attributes that impact the immunogenicity of a protein include its primary amino acid sequence, chemical degradability, and its propensity for misfolding.^[^ ^48^, ^49^ ^]^ However, in general, there is a need for better predictive tools that can account for the heterogeneity of patients and treatments to advance the understanding between PNP properties and potential immunogenicity.^[^ ^47^, ^48^ ^]^

The high degree of flexibility of engineering PNPs underscored by the range of possible proteins that can be used as well as the technologies in place to produce them. The common methods to produce PNPs include nab‐technology, emulsification, desolvation, self‐assembly, nanospray drying, and electrospraying.

## 2 Nanoparticle Albumin Bound Technology

The nanoparticle albumin bound (Nab) process produces albumin nanoparticles capable of encapsulating hydrophobic drugs through a combination of high shear, cavitation, and pressure. High pressure homogenizers force water insoluble or partially soluble drugs into the albumin solution. Although other proteins have been considered, albumin is the ideal candidate due to its 35 cysteine residues ‐34 are occupied through disulfide linkages, and only one Cys residue features a free sulfhydryl group (Cys34).^[^ ^50^ ^]^ When the crude mixture is introduced to high shear conditions, the disulfide bridges are disrupted, and under further involvement of Cys34 are reshuffled into new disulfide bonds. If needed, chemical modification can be employed to introduce additional Cys residues.

Nab technology has been successfully applied to a range of small‐molecule drugs. Among the most successful are taxanes, such as paclitaxel and docetaxel. Taxanes represent a particularly important class of antineoplastic drugs which work by stabilizing microtubules and preventing proliferation. Traditional methods for delivering these agents require solubilization in surfactants to improve their bioavailability and solubility, but also introduce adverse side effects and cumbersome treatment regimens. For the solvent‐based delivery of paclitaxel for example, mixtures of Cremophor EL (CrEL) and ethanol must be prepared. This procedure is associated with a range of detrimental effects, such as the fact that CrEL can cause leaching of plasticizers from medical‐grade polyvinyl chloride tubing sets and elevates hepatic toxicities.^[^ ^51^ ^]^ Further, CrEL is known to cause hypersensitivity reactions.^[^ ^52^, ^53^ ^]^ Outside of toxicities and administration issues, the conventional CrEL delivery displays non‐linear pharmacokinetics in the case of paclitaxel which has been attributed to the formation of CrEL micelles, which entrap paclitaxel and reduce the amount of free drug available.^[^ ^54^ ^]^

In 2005, American Bioscience Inc. developed Nab paclitaxel, one of the very first nanomedicines that has subsequently been approved by the FDA and commercialized under the tradename Abraxane for the treatment of metastatic breast cancer. These PNPs are formed by high shear in a high‐pressure homogenizer optimized to form ≈130 nm sized paclitaxel loaded albumin nanoparticles.

**Figure 1:**
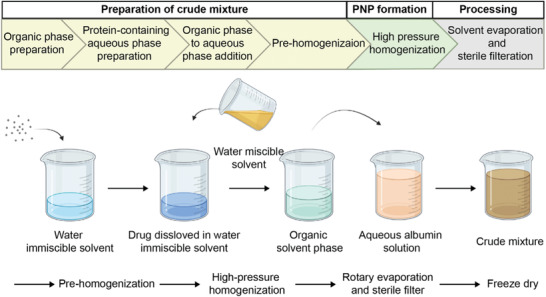
Workflow illustration of nanoparticle albumin bound (Nab) technology. Created with BioRender.com.

**Figure** **1** depicts the main steps of the Nab technology. The albumin PNPs are produced by first dissolving the paclitaxel in a water immiscible organic solvent (<5% solubility in water) to obtain an “oily phase”.^[^ ^55^ ^]^ A water miscible organic solvent (>10% solubility in water) is added to create the organic phase mixture.^[^ ^55^ ^]^ This organic phase mixture is reconstituted into an emulsion comprised of an aqueous protein phase and an organic phase.^[^ ^55^ ^]^ Unlike in direct emulsification, the use of external surfactants is not necessary during Nab technology. This crude mixture is first subjected to an initial, pre‐homogenization then added to a high‐pressure homogenizer where it experiences high local shear, cavitation, and local heating thereby forming new intermolecular crosslinks within the protein.^[^ ^55^ ^]^ The remaining solvent is removed and the <200 nm PNPs are isolated by filtration.

Exploiting albumin's ability to naturally sequester hydrophobic molecules enables Nab‐paclitaxel formulations to simultaneously overcome several drawbacks associated with the traditional methods.^[^ ^13^ ^]^ Not surprisingly, Abraxane was also FDA approved for first line treatment of advanced non‐small cell lung cancer and late‐stage pancreatic cancer in 2012 and 2013, respectively. More recently (2019), Abraxane in combination with Tecentriq (a PD‐L1 inhibitor) was approved for advanced triple negative breast cancer expressing PD‐L1. In a recent meta‐analysis of clinical trials into metastatic breast cancer, Nab paclitaxel was significantly better than conventional approaches in overall response rate, disease control rate, progression‐free survival, and overall survival with the adverse events and dose discontinuation rate being comparable between both methods.^[^ ^56^ ^]^ Abraxane is continuously being expanded to other cancers, particularly as a combination therapy, to augment current treatments or as a maintenance treatment.^[^ ^57^, ^58^, ^59^ ^]^ According to clinicaltrials.gov, 302 clinical trials have been completed with using the search key of Nab paclitaxel or Abraxane. There are 135 active clinical trials with Nab paclitaxel and 266 studies that are recruiting.

Although other instruments can induce high shear and cavitation as shown in **Table** **2**, high pressure homogenization remains the method of choice. High pressure homogenizers work by passing the initial material through one small orifice (one stage) or two small orifices (two stage) under high pressure (typically 10–500 MPa) and thereby facilitating size reduction through a combination of shear, cavitation, and turbulence.^[^ ^60^ ^]^ Both the pressure and number of cycles in high pressure homogenizers have been studied as important parameters affecting particle size.^[^ ^60^ ^]^ In addition to the homogenization parameters, other parameters including the choice of organic solvent to solubilize the therapeutic agent, the ratio of drug solution to aqueous HSA solution, and concentration of the drug have shown to influence the size and its distribution.^[^ ^61^ ^]^ Furedi et al. found when optimizing a voriconazole‐loaded albumin nanoparticle, increasing the cycles to 6 or above resulted a stable polydispersity index.^[^ ^61^ ^]^

**Table 2:** Process parameters and alternative instrumentation for Nab technology^[^ ^55^ ^]^

Component/instrument	Criteria	Examples
Water immiscible organic solvent	Water immiscible organic solvent (usually < 5% solubility in water)	Chloroform
Water miscible organic solvent	Water miscible organic solvent (>10% solubility in water)	Ethanol, ethyl acetate, tetrahydrofuran, dioxane, acetonitrile, butanol, acetone, glycerol, propylene glycol, dimethyl sulfoxide, dimethyl formamide, methyl pyrrolidinone
Stabilizing agent	Stabilizing agent that contains free sulfhydryl groups and/or disulfide linkages which may be introduced via chemical modification.	Albumin, immunoglobulin, casein, insulin, hemoglobin, lysozyme, fibronectin
Instrument for inducing shear	Must produce high shear and cavitation	High pressure homogenization, high shear mixers, sonication, high shear impellers
Evaporation methods	Remove solvents	Rotary evaporator, falling film evaporator, spray driers, freeze driers, ultrafiltration
Drying methods	Obtain product as a powder	Freeze drying, spray drying

Since the development of Abraxane, others have prepared PNPs though high‐pressure homogenization encapsulate therapeutics to reduce toxicity associated with traditional delivery, improve bioavailability, and/or alter the pharmacokinetics. While many are interested in delivering active agents as shown in **Table** **3**, stimuli responsive drugs like photosensitizers for photodynamic therapy can also be incorporated.

**Table 3:** Studies of Nab‐technology produced nanomedicines

Application	Protein	Drug/loaded entity	Cycles	Pressure [MPa]	Solvent	Size [nm]	Refs.
Anti‐fungal treatment	Albumin	Voriconazole	6	≈179	Chloroform	35‐85	[61]
Idiopathic pulmonary fibrosis	Albumin	Tacrolimus	9	138	Chloroform and ethanol	182.1 ± 28.5	[62]
Pancreatic cancer	Albumin	Gemcitabine	9	138	Chloroform	150 ± 27	[63]
Colon cancer, pancreatic carcinoma	Albumin	Curcumin	9	138	Chloroform	130–150	[64]
Non‐small cell lung cancer	Bovine serum albumin	Curcumin	9	138	9:1 Chloroform: Ethanol	128.3 ± 3.0	[65]
		Curcumin and doxorubicin				134.0 ± 14.7	
		Doxorubicin				131.80 ± 8.4	

Specifically, Temoporfin (mTHPC), a photosensitizer, was incorporated into the Nab system to mitigate prolonged infusion rates and adverse side effects with the commercially available Foscan, which is formulated with ethanol and propylene glycol.^[^ ^66^ ^]^

In summary, Nab technology is a fabrication method of albumin nanoparticles encapsulating hydrophobic drugs through a combination of high shear, cavitation, and pressure. Nab technology is the first commercialized nanomedicine (Abraxane) after FDA approvement in 2005. One of advantages of Nab technology is a delivery of hydrophobic drugs without incorporating harmful solubilizers such as CrEL. Nab technology overlaps with some aspects of the emulsification procedure, as it can be thought of as a contemporary emulsification approach, with the difference that the Nab technology does not require external crosslinking to ensure stability, the use of surfactants is not necessary, and there are more degrees of control within the process.^[^ ^67^ ^]^ Potential drawbacks of this technique include limitations on the stabilizing agents if they do not naturally contain or cannot be chemically modified to have sulfhydryl and/or disulfide groups. However, it cannot be overlooked that this method has enjoyed broad translational successes.

## 3 Emulsification Methods

Emulsions are defined as mixtures of two immiscible liquid phases driven by mechanical shear and stabilized through surface energy lowering agents (surfactants).^[^ ^68^, ^69^ ^]^ The type of instrument to impart mechanical shear along with the type of surface reducing agent depends on emulsification method. The choice of emulsification method is dictated by the application of interest, which can range from food manufacturing to drug delivery design.^[^ ^70^, ^71^ ^]^

Various types of emulsions exist and are defined according to the composition of the continuous phase, the order of addition and even the type of surface reducing agents. The simplest type of emulsions are single emulsions like water‐in‐oil (W/O) and oil‐in‐water (O/W). Droplets of immiscible dispersions inside a continuous phase are thermodynamically unstable and require additional molecules to reduce the surface energy of the interface. Surface reducing agents such as surfactants or otherwise referred to as emulsifiers, are critical to lower the energy between the two phases which are susceptible to coalescence. Acting as a barrier between the two phases, relatively stable nanoparticles can be formed. The degree of stability, influence on emulsifiers in particular emulsification systems, and mechanisms behind aging of emulsions has been reviewed elsewhere.^[^ ^72^, ^73^, ^74^ ^]^

To produce PNPs via emulsification, W/O methods are exclusively reported. Briefly, the immiscible solution is exposed to low speed homogenization to incorporate both phases and induce mechanical shear. When added dropwise to an oil phase in the presence of a surfactant, particles are produced. While a surfactant is used to produce the PNPs by reducing the surface tension between the aqueous protein solution and the oily phase, PNPs need external crosslinking once the PNPs are produced. Two methods are commonly used: the addition of chemical crosslinkers and heat denaturation.

**Figure 2:**
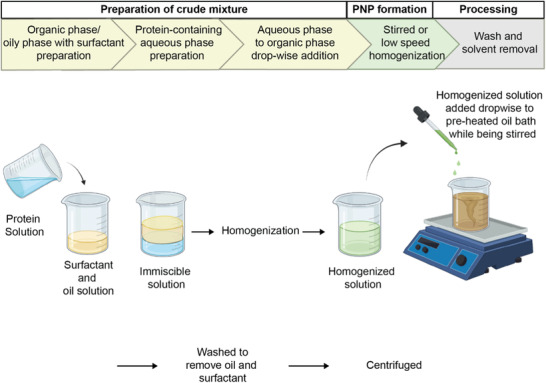
Workflow illustration of preparing albumin nanoparticles through heat denatured water in oil emulsification. Created with BioRender.com.

The emulsification of proteins was first reported in 1972 by Scheffel and coworkers.^[^ ^75^ ^]^ Albumin microspheres were produced by homogenizing cottonseed oil with HSA. These particles were thermally stabilized by adding the homogenized mixture to a preheated oil bath dropwise. The mixture was then cooled, centrifuged, and washed with ethyl ether to aid in the removal of excess oil through centrifugation. The PNPs were further processed by passing them through a 0.22 µm filter and finally dried under UV light. Since then, others explored this method to produce albumin microspheres to optimize the process, understand the parameters, and load the particles with therapeutic agents. The trend for investigating the preparation of albumin microspheres appeared to be on an upward trend in the 1980's, and the expansion of this technique to produce nanoparticles would be expected.^[^ ^76^, ^77^, ^78^, ^79^, ^80^ ^]^ However, first reports of nanoscale particles using this method only emerged in the mid‐2000's, when Yang and coworkers synthesized hydroxycamptothecin (HCPT) loaded BSA nanoparticles using a single emulsion (water‐in‐oil) for cancer applications and is illustrated in **Figure** **2**.^[^ ^81^ ^]^ This method to produce albumin nanoparticles displays similarities of the preparation of albumin microspheres conducted by Gallo et al.^[^ ^76^ ^]^ The HCPT was combined with BSA in sodium hydroxide then added to an oil phase. The immiscible phases were emulsified using a homogenizer then added dropwise to a pre‐heated castor oil bath at 140 °C while being mixed. The solution was cooled and washed with petroleum ether and centrifuged to remove excess oil. The resulting PNPs were ≈600 nm in size. Parameters of the method were investigated in order from BSA concentration, high speed emulsification time, aqueous to non‐aqueous phase volume ratio, emulsion drop rate, heat stabilization temperature and heat stabilization time.^[^ ^81^ ^]^ It was found that an increase in BSA concentration decreases the particle diameter as well as an increase in emulsification speed and the lowest aqueous to non‐aqueous phase volume ratio. The HPCT loading, encapsulation efficiency and cumulative release was 2.21%, 57.5%, and 25%, respectively.

Crisante and coworkers prepared BSA nanoparticles through a W/O emulsion chemically crosslinked with glutaraldehyde and with the antibiotic, cefamandole, adsorbed.^[^ ^82^ ^]^ The aqueous phase consisted of BSA and glutaraldehyde while the organic phase consisted of cyclohexane with Span80. The aqueous phase was added dropwise to the organic phase, homogenized, centrifuged then washed with deionized water. Isopropanol was used to remove excess Span80 and glutaraldehyde. Cefamandole was adsorbed on the BSA nanoparticles through dialysis. The final BSA nanoparticles were ≈430 nm in size and could adsorb 20% of cefamandole. It was found that BSA solubilized in an aqueous solution of a pH close to its isoelectric point (pH 5.3), caused the PNPs to be larger likely due to aggregation.^[^ ^82^ ^]^ To optimize drug loading and release, BSA nanoparticles with cefamandole entrapped in a polyurethane matrix was also investigated. It was found that the polyetherurethane acid was capable of adsorbing 5 times more cefamandole than BSA nanoparticles without polyetherurethane acid. The matrix acted as a diffusion barrier which prolonged the release and showed antimicrobial activity for up to 8 days.^[^ ^82^ ^]^

Emulsification has been widely explored in the context of polymeric nanoparticle development where various types of emulsions like double emulsions, pickering emulsions, and microemulsions have been investigated. However, the breadth and depth of emulsification has not been applied to PNP development due to the amphipathic nature of proteins enabling them to act as surfactants themselves to reduce the surface tension in emulsification. In fact, PNPs are routinely used in applications like food to stabilize the water and oil interface in pickering emulsions where droplets are susceptible to coalescence.^[^ ^70^, ^83^, ^84^ ^]^ The advantage using nanoparticles as surface reducing agents as opposed to other surfactants is that they are able to more strongly adsorb to the oil‐water interface thereby achieving greater stability.^[^ ^85^ ^]^ A variety of methods are available to produce these PNPs as emulsifiers in aid of emulsification of other materials of interest; however, PNP production through emulsification techniques is very limited and arguably the least common method in PNP development.^[^ ^70^ ^]^

Beyond water‐in‐oil emulsification, there has not been substantial progress with producing PNPs via emulsification. This can be attributed to a few reasons. The first is that proteins themselves act as emulsifiers because of their amphiphilic nature. They are such effective stabilizers, PNPs are routinely used in food applications. Therefore, the role of proteins in emulsification is better suited as a component of the process than the desired product. The second reason is that there are other methods available that produce more desirable properties like higher loading efficiency and smaller size. A contemporary method, Nab technology, has made PNP synthesis through emulsification somewhat obsolete. Nab technology addresses drawbacks associated with emulsification; it eliminates the requirement of external crosslinking and the use of surfactants. Simultaneously, this method can achieve smaller particle distributions than reported for emulsification. **Table** **4** compares the two techniques according to their merits, demerits along with critical processing parameters that influence their size.

**Table 4:** Comparison of protein‐based nanoparticle synthesis methods

Technique	Merits	Demerits	Critical parameters affecting nanoparticle size	Refs.
Emulsification	Only requires typical laboratory equipment	Relatively large nanoparticles Low loading Release of drug is difficult to control Removal of surfactant and oil	Concentration Emulsification time Emulsifier Ratio of aqueous to non‐aqueous solutions pH of aqueous solutions	[81, 82]
Nab‐technology	No external crosslinking methods needed Entrapment of hydrophobic drugs	Relies on presence of sulfhydryl and/or disulfide groups for crosslinking	Choice of organic solvent Concentration of drug Ratio of drug to protein solutions Concentration of protein Number of cycles of the HPH Pressure of HPH	[60, 61]
Desolvation	High yield Easy to manufacture Low cost Narrow dispersity of PNPs	Possible protein denaturation leading to loss of function	Desolvating agent amount pH of protein solution Desolvating agent dielectric constant Desolvating agent addition mode	[86, 87, 88, 89, 90]
EHD	Narrow dispersity of PNPs Retainment of secondary structure Multicompartment PNPs Ability to entrap both hydrophobic and hydrophilic drugs PNPs in dry state (avoiding degradation or undesired payload release)	Low throughput Molecular weight dependent Lack of spatial control of payload	Protein concentration Solvent dielectric constant Macromer to protein ratio Crosslinker properties	[91, 92, 93, 94]
Self‐assembly	Monodisperse particles Unlimited geometry of PNP Unlimited monomeric peptide design space	Hinges upon computational calculations Low yield Unavailable for existing native proteins	Protein concentration Monomeric peptide design	[33, 95, 96, 97, 98, 99, 100, 101, 102, 103, 104, 105]

In summary, emulsification is a fabrication of protein emulsions by using immiscible solvents with interfacial stabilizer, followed by crosslinking and purification. This technique is a comprehensive fabrication method, and various proteins can be applied. Although resulting particle size is relatively large (≈430 nm) with low loading efficiency (≈2.21%), the fact that only typical laboratory equipment is necessary for the operation makes this technique convenient and accessible.

## 4 Desolvation Methods

**Figure 3:**
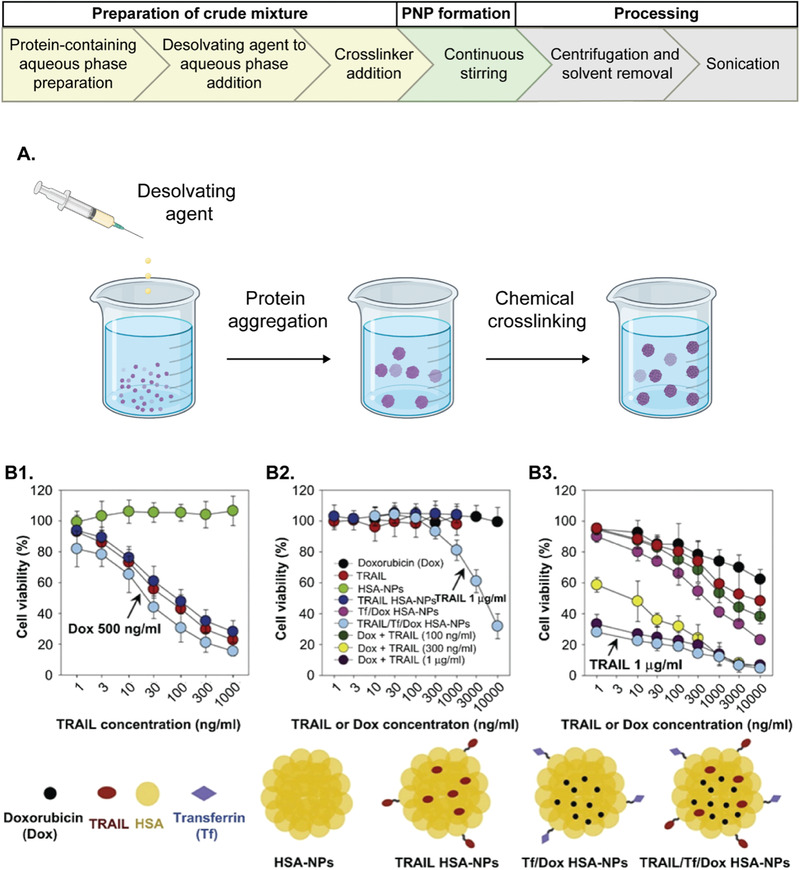
PNPs fabricated by desolvation method. A) Desolvation workflow. Created with BioRender.com. B1–B3) Cytotoxicity data as measured by MTT assays. B1) Cytotoxicities of blank HSA‐NPs, TRAIL, TRAIL HSA‐NPs, and TRAIL/Tf/Dox HSA‐NPs (Dox concentration at 500 ng mL^−1^) on HCT 116 cells; B2) Cytotoxicities of Dox, TRAIL, TRAIL HSA‐NPs, and TRAIL/Tf/Dox HSA‐NPs (TRAIL conc. 1 µg mL^−1^) on MCF‐7/ADR cells; B3) Cytotoxicities of Dox, TRAIL, Dox + TRAIL (100, 300, 1000 ng mL^−1^), Tf/Dox HSA‐NPs, and TRAIL/Tf/Dox HSA‐NPs (TRAIL concentration at 1 µg mL^−1^) on CAPAN‐1 cells. Reproduced with permission.^[^ ^113^ ^]^ Copyright 2012, Elsevier.

Desolvation is one of the more frequently used fabrication methods to produce PNPs.^[^ ^106^, ^107^ ^]^ Nanoparticle fabrication relies on precipitation in the presence of a desolvating agents, that is, a poor solvent, to reduce the protein's water‐solubility to induce aggregation.^[^ ^107^ ^]^ Exposure to a desolvating agent induces conformational changes in the protein and decreases its overall solubility.^[^ ^107^ ^]^ Once a threshold is reached, the protein undergoes phase segregation and precipitates in the form of PNPs.^[^ ^106^, ^107^, ^108^ ^]^ However, PNPs still can lack permanent stability and can rapidly undergo redissolution, requiring the need for a secondary treatment to induce crosslinking (**Figure** **3**A).^[^ ^106^ ^]^ Owing to its simplicity, scalability, and low fabrication costs, desolvation methods are frequently employed for PNP production.^[^ ^89^ ^]^

Choice of process and solution parameters for the desolvation method can influence the size and physicochemical properties of the resulting PNPs (**Table** **5**). Weber et al. systematically characterized the effects of desolvating agent and crosslinking method on size, zeta potential, and the number of available surface‐amine groups of HSA PNPs.^[^ ^86^, ^87^ ^]^ The addition of the desolvating agent, ethanol, resulted in an increase in the particle size until the ethanol volume exceeded a threshold of 150% of the initial volume, whereafter further addition of desolvating agent led to an increase in the number of particles while the particle size remained the same. Crosslinking by glutaraldehyde or heat denaturation critically influenced the amount of accessible amino groups on the particle surfaces and weakly altered their zeta potential, whereas no significant effect on particle sizes was observed.^[^ ^86^ ^]^ Not surprisingly, heat denaturation‐stabilized HSA nanoparticles contained higher number of surface amino groups compared to glutaraldehyde stabilization.^[^ ^86^ ^]^ However, the heat denaturation temperature and treatment duration had no effects on either particle sizes or number of amino groups on the particle surfaces.

**Table 5:** The influence of different desolvation method process parameters on PNPs size

Parameter	Parameter variation	Influence on size	Ref.
Desolvating agent amount	500–150 (% of initial volume)	Increase	[86]
Glutaraldehyde concentration	0–200%*	No effect	[86]
Heat denaturation time	2–48 [h])	No effect	[86]
Heat denaturation temperature	50–70 [°C]	No effect	[86]
pH of HSA solution	7–9	Decrease	[87]
Ethanol addition rate	0.5–2 [mL min^−1^]	No effect	[87]
HSA concentration	25–100 [mg mL^−1^]	No effect	[87]
Desolvating agent dielectric constant	20.7–78.3	Decrease	[88]
Stirring rate	500–800 [rpm]	No effect	[88]
Pre‐stirring time	0–4 [h]	No effect	[88]
Desolvating agent addition mode	Dropwise to continuous	Decrease	[88]
Imidazole concentration	0–250 [mm]	Decrease	[90]

* Percentage of the theoretic glutaraldehyde amount required to crosslink entire lysine groups in the system (there are 59 lysine residues in each HSA molecule)

In a follow‐up study, more subtle effects, such as the addition rate of the desolvating agent or the composition of the HSA solution and its pH value were explored (Table 5).^[^ ^87^ ^]^ Among the parameters studied, the pH of the HSA solution prior to the desolvation procedure was the main factor impacting the particle size. Increasing pH reduced the diameter of PNPs; at higher pH values (pH > 9), PNPs had diameters of about 150 nm. Subsequent purification by differential centrifugation resulted in narrowly distributed PNP populations.^[^ ^87^ ^]^

Storp et al. investigated particle size distributions with respect to desolvating agents with differing dielectric constants, stirring rates, and pre‐stirring times of the HSA (Table 5).^[^ ^88^ ^]^ The study showed that a stirring rate of at least 500 rpm and continuous addition of desolvating agent led to smaller and more monodisperse PNPs.^[^ ^88^ ^]^ Among non‐solvents used in the desolvation method,^[^ ^88^ ^]^ ethanol is one of the most common desolvating agents.^[^ ^86^, ^87^ ^]^ Increasing the concentration of ethanol and acetone results in an increase in particle size, whereas the methanol concentration did not influence PNPs sizes in the same manner (**Table** **6**). Ultimately, a correlation between dielectric constant and the PNPs size was observed where higher dielectric constants led to smaller particles.^[^ ^88^ ^]^ In general, particles fabricated by desolvation methods tend to be more polydisperse and larger than comparable PNPs made by other methods such as self‐assembly or electrospraying.^[^ ^89^ ^]^

**Table 6:** The influence of desolvating agents’ type and concentration on PNPs size

Solvent	Solvent concentration [m m%^−1^]	Size [nm]
Acetone	70–90	140–300
Ethanol	80–95	150–200
Methanol	80–100	55–60
Ethanol and methanol	30–90 [ethanol]	60–140

While organic solvents are typically used as desolvating agents, aqueous solutions with high salinity can also be employed for PNPs preparation.^[^ ^107^ ^]^ As the salt concentration is increased, the electrostatic interactions between proteins are screened, causing aggregation and precipitation.^[^ ^109^ ^]^ This approach, often referred to as salting‐out method, is a relatively simple approach that avoids adverse conformational changes that may impact the activity of the protein.^[^ ^107^ ^]^ This technique was used to fabricate insulin nanoparticles with tunable sizes between 100 and 1600 nm in a pH dependent manner.^[^ ^110^ ^]^ At sodium chloride concentrations > 0.55 m, insulin precipitated in the form of PNPs.^[^ ^110^ ^]^ However, when the salt concentration was too high (>0.8 m), the solubility of nonpolar groups on the insulin particle surfaces was decreased and therefore enhanced hydrophobic interactions between the particles were observed which resulted in larger particle aggregates.^[^ ^110^ ^]^ In another example, silk PNPs were prepared via the salting‐out method with potassium phosphate. Silk PNPs had controllable sizes ranging from 500 to 2000 nm and tunable secondary structures.^[^ ^111^ ^]^

As outlined in Figure 3A, desolvated PNPs require a crosslinking step to ensure their stability and to avoid rapid dissolution.^[^ ^107^ ^]^ Details about strategies to stabilize PNPs will be discussed in Chapter 7, however, one of the most common methods involves glutaraldehyde crosslinking whereby the functional groups of the protein (i.e., amine, thiol, phenol, and imidazole) form covalent bonds with aldehyde groups.^[^ ^90^, ^112^ ^]^ During this process, a significant proportion of the amine groups is consumed due to the chemical conjugation with glutaraldehyde.^[^ ^113^ ^]^ To ensure sufficiently high concentrations of interfacial amine groups on PNPs, protection strategies have been employed. For example, dimethylmaleic anhydride was used to protect surface‐bound amine groups and to enable subsequent modification of PNPs with targeting ligands such as tumor necrosis factor (TNF)‐related apoptosis‐inducing ligand (TRAIL) or even transferrin (Tf).^[^ ^113^ ^]^ This strategy has been successfully implemented for Dox‐loaded HSA PNPs that featured loading efficiencies of about 95% and particle sizes of about 220 nm. The cytotoxicity of the components, TRAIL, doxorubicin (Dox) and various HSA PNP control groups were evaluated in three cancer cell lines and in particular the fully formulated TRAIL/Tf/Dox HSA‐PNPs showed high activity against several cancer cell lines in in vitro experiments (Figure 3B1).^[^ ^113^ ^]^ Importantly, the TRAIL/Tf/Dox HSA‐PNPs also displayed significant activity against Dox‐resistant cells which clearly distinguished them from control groups loaded with only Dox (Figure 3B2).^[^ ^113^ ^]^ Under certain conditions, either of the two components, TRAIL or Dox, alone were not effective, whereas the binary combination showed highly synergistic effects (Figure 3B3).^[^ ^113^ ^]^

Although chemical crosslinking agents, such as glutaraldehyde, have been often used to stabilize PNPs, this strategy is plagued by potential cytotoxicity due to residual aldehyde groups. Thus, alternate chemical modification routes should be considered for in vivo applications. To mitigate the cytotoxicity of glutaraldehyde, a natural amine reactive crosslinker, genipin, can be used to stabilize proteins, such as recombinant human gelatin nanoparticles.^[^ ^114^ ^]^ While the crosslinking time for genipin is longer compared to glutaraldehyde, the cytotoxicity is ≈10 000× lower.^[^ ^114^ ^]^

As an alternative approach to the permanent chemical crosslinking, reversible crosslinkers such as dithiobis succinimidyl propionate have found increased attention.^[^ ^115^, ^116^ ^]^ While these crosslinkers are cleavable under certain biological environments, they still may result in an alteration of the original protein structure.^[^ ^117^ ^]^ In contrast, reversible disulfide‐based cross‐linkers, such as dithio‐bis(ethyl *1H*‐imidazole‐1‐carboxylate), still allow for rapid intracellular degradation of PNPs due to the reducing milieu of the cytoplasm, yet leave the original proteins unaltered after disulfide cleavage.^[^ ^118^ ^]^ Alternatively, redox‐responsive PNPs have also been prepared without any crosslinkers. For example, the disulfide groups of HSA have been reduced in an additional pre‐modification step, prior to desolvation.^[^ ^117^ ^]^ Glutathione, one of the major endogenous antioxidants in vivo, is a probate reducing agent to break up the intramolecular disulfide bonds within the native HSA. After desolvation of reduced HSA, new disulfide bonds spontaneously formed and 110–190 nm PNPs were isolated that were already stabilized and were void of any toxic exogenous chemical crosslinkers.^[^ ^117^ ^]^ Turbidity measurements showed that these self‐crosslinked nanoparticles gradually dissolve in the reducing environment, while no change was observed in the absence of a reducing agent.^[^ ^117^ ^]^ Apart from chemical crosslinking, the stabilization of desolvated PNPs has been achieved by adsorption of a chitosan shell via electrostatic self‐assembly. Strong electrostatic attraction between negatively charged protein and positively charged chitosan effectively stabilized desolvated PNPs.^[^ ^119^ ^]^ Using this approach, albumin PNPs loaded with the NEL‐like molecule‐1 (NELL‐1) protein were prepared.^[^ ^119^ ^]^ If chemical crosslinking is not possible, ionizing radiation provides an alternative for stabilizing desolvated PNPs.^[^ ^120^, ^121^, ^122^ ^]^ For example, the model protein papain was stabilized via radiation‐induced crosslinking and resulted PNPs ranging from 11 to 900 nm.^[^ ^122^ ^]^

While desolvation is a popular, simple, and cost‐effective technique to produce PNPs, it requires the use of organic solvents and/or crosslinking strategies that alter the protein's native structure and therefore can lead to a loss of function and increased immunogenicity.^[^ ^89^, ^90^ ^]^

In summary, desolvation, a commonly used PNP fabrication strategy, works by decreasing the solubility of protein in solution through desolvating agents enabling conformation changes within the protein and ultimately leading to precipitated nanoparticles. The disadvantage of this technique is that external crosslinking is necessary to ensure stability which can alter the structure of the native protein leading to a loss of function, immunogenicity, and poor drug loading; however, the strategies to induce stability range from physical methods (heat denaturation, ionizing radiation) to chemical crosslinking (glutaraldehyde, genipin). Advantages of desolvation‐produced PNPs include low cost, high throughput, and a high degree of flexibility in process parameters to achieve particular nanoparticle properties (i.e., size, surface charge, stimuli‐responsiveness) (Table 4). Despite the disadvantages associated with desolvation to produce PNPs, it will likely remain a staple fabrication technique for PNPs as more modifications to processing parameters are investigated.

## 5 Liquid Atomization Methods

### 5.1 Nanospraying

Spray drying is a well‐established process commonly used in the pharmaceutical, chemical, and food industries to produce dry powder from a liquid phase in a one‐step continuous process.^[^ ^123^ ^]^ This technique is comprised of a four‐stage setup: i) atomization of the input solution into a spray, ii) spray‐air (hot drying gas) contact, iii) drying of the spray, and iv) separation of the dried final product from the drying gas.^[^ ^123^, ^124^ ^]^ The particle properties can be tuned by manipulation of the process parameters and are impacted by the particular spray dryer configuration. The feed solution is atomized into a spray of fine droplets, which is then brought into interaction with the hot drying gas leading to moisture evaporation and solid particles formation.^[^ ^124^ ^]^ Conventional spray dryers suffer from a low yield of separation and collection of fine particles < 2 µm.^[^ ^125^, ^126^ ^]^ However, technological advancements related to spray drying components, such as spray head, heating system, and particle collector system have enabled the preparation of PNPs.^[^ ^124^ ^]^ The latest generation of spray dryers, such as the Nano Spray Dryer B‐90, take advantage of piezoelectric‐driven vibrating mesh technology.^[^ ^124^, ^125^ ^]^ In this case, the spray head features a piezoelectric crystal that is mechanically coupled with a thin perforated membrane. This membrane, also referred to as spray mesh, is comprised of an array of micron‐sized holes.^[^ ^125^ ^]^ When the piezoelectric actuator is driven at an ultrasound frequency, it initiates oscillating mesh vibrations leading to the production of an aerosol that contains millions of monodisperse droplets.^[^ ^125^ ^]^ Lee et al. investigated the applicability of the Nano Spray Dryer B‐90 for the fabrication of BSA nanoparticles.^[^ ^124^ ^]^ The effect of different process parameters (spray mesh size, inlet temperature, and drying gas flow rate) and solution parameters (protein and surfactant concentration) were studied on the particle properties such as size and morphology.^[^ ^124^ ^]^ The results showed that spray dry mesh size and surfactant concentration were the major factors influencing the particle size and morphology, respectively.^[^ ^124^ ^]^ Using the 4 µm spray mesh, 0.05% surfactant, and 0.1% BSA concentration resulted in the production of 460 nm spherical BSA nanoparticles.^[^ ^124^ ^]^

Overall, various industries employ nanospray drying as it is a relatively streamlined, hands‐off method to produce solid protein‐nanoparticles from an initial aqueous formulation input. This technique relies on the spray dryer, which has witnessed a few generations of improvement to obtain smaller, monodisperse particle populations and higher throughput. Aside from this technique requiring less user involvement than others, it has also shown that the design space for improvements to the spray dryer instrumentation continues to be improved upon, suggesting potential growth of this method. Since there are limited studies using this method to fabricate PNPs along with recent improvements to the instrumentation, it is difficult to assess how it will fare when compared to the other methods. Its success will likely depend on whether this technique can produce particles with characteristics competitive with the other methods to warrant its use. For example, most nanomedicines are <460 nm, so if size cannot be changed, it may render this technique less useful for PNP fabrication.

### 5.2 Electrospraying

During atomization via electrosprays, electrical potential differences of several kilovolts are employed to disperse a fine mist of nanodroplets.^[^ ^127^, ^128^ ^]^ The solution at the tip of the capillary experiences the electric field causing positive ions to accumulate at the liquid surface. Electric shear stresses distort the meniscus to establish the Taylor cone.^[^ ^128^, ^129^, ^130^ ^]^ With increasing the voltage, the repulsive electrostatic forces overcome the surface tension, and the jet breaks up into small droplets.^[^ ^127^, ^128^, ^130^ ^]^ The highly charged nanodroplets travel towards the grounded collection plate. During this process, the solvent is rapidly evaporated and the remaining non‐volatile compounds are solidified into micro‐ or nanoparticles.^[^ ^128^, ^130^ ^]^

Gomez et al. evaluated the applicability of electrospraying to produce monodisperse and biologically active insulin PNPs.^[^ ^131^ ^]^ Insulin was dissolved in an acidified ethanol:water:hydrochloric acid (HCl) solvent system.^[^ ^131^ ^]^ The solution was then electrosprayed and formed doughnut shaped PNPs with sizes ranging from 98 to 117 nm.^[^ ^131^ ^]^ Decreasing the insulin concentration and/or lowering the liquid flow rate created even smaller PNPs. At higher flow rates, polydisperse PNPs were observed.^[^ ^131^ ^]^ Compared to the control insulin, the electrosprayed insulin PNPs showed no difference in insulin receptor binding properties.^[^ ^131^ ^]^ Electrospraying has also been employed to prepare *β*‐carotene loaded whey PNPs with an encapsulation efficiency of 90% for food applications.^[^ ^132^ ^]^ In this system, the size of nanoparticles was tunable by controlling the pH value of the protein solution.^[^ ^132^ ^]^ The fact that electrospraying doesn't require the use of organic solvents or external heating are critical features when considering PNPs for health applications, because it eliminates potential toxicity issues and avoids the destruction of sensitive bioactivities.^[^ ^132^ ^]^ Cardoso et al. utilized electrospraying to formulate PNPs with a commonly used excipient for pulmonary delivery, lactose.^[^ ^133^ ^]^ When dimethyl sulfoxide (DMSO) was used in the electrospraying process, a thin surface layer of protein was recovered, whereas using ethanol as solvent led to the formation of 700 nm particles.^[^ ^133^ ^]^ Circular dichroism (CD) spectroscopy measurements demonstrated that the process did not cause any significant denaturation or conformational changes to the protein, indicating its potential as a pulmonary delivery platform for therapeutic proteins.^[^ ^133^ ^]^

### 5.3 Electohydrodynamic Co‐Jetting

Electrohydrodynamic (EHD) co‐jetting has been used to prepare more complex particles including multicompartmental micro‐ and nanoparticles with various applications in drug delivery.^[^ ^134^, ^135^, ^136^, ^137^, ^138^, ^139^, ^140^, ^141^, ^142^, ^143^, ^144^, ^145^, ^146^ ^]^ EHD co‐jetting utilizes two or more needles as capillaries in a side‐by‐side configuration. The input solutions are pumped into the needles at a flow rate forming laminar flow to ensure a stable interface between the jetting solutions. Once a droplet forms out of the needle, an electric field is applied to the system that distorts the meniscus into a Taylor cone and forms an electrified polymer jet. This jet breaks up into a spray of fine, charged droplets that undergo rapid solvent evaporation and subsequent solidification of nonvolatile components. Due to rapid solvent evaporation, the initial flow‐determined geometry of the input solutions is preserved in the fabricated nanoparticles giving access to particle architectures that would be otherwise impossible or hard to achieve.

The size of the nanoparticles is tunable by changing the EHD jetting parameters that are either jetting solutions‐related (surface tension, dielectric constant, viscosity, polymer concentration, and molecular weight), process‐related (fluid flow rates, the distance between the needle and a counter electrode, the electric voltage, and the capillaries diameter) or environment‐related (temperature, pressure, and humidity).^[^ ^147^ ^]^

**Figure 4:**
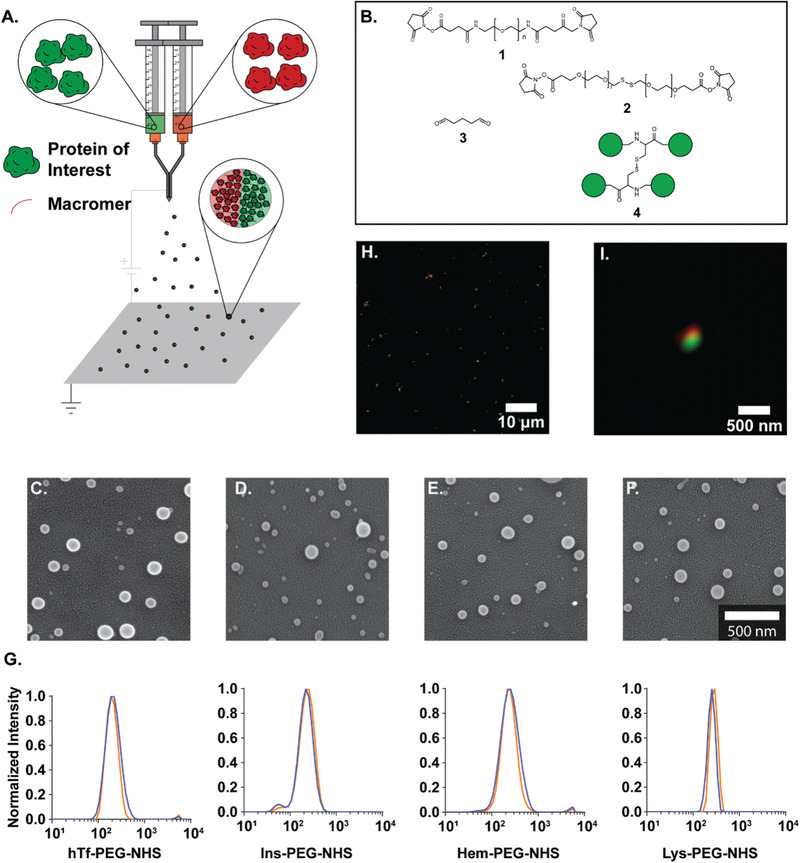
Electrohydrodynamic jetting can be used to fabricate PNPs from different proteins. A) Schematic illustration of EHD co‐jetting setup. B) Different macromer used to stabilize EHD jetted PNPs. Scanning electron microscopy (SEM) images of PNPs prepared from C) human transferrin, D) insulin, E) hemoglobin, and F) lysozyme. G) Dynamic light scattering (DLS) measurements of PNPs after 7 days storage at 4 °C. H) SIM microscopy images of bicompartmental PNPs. I) Zoomed‐in image of an individual bi‐compartmental PNP. Reproduced with permission.^[^ ^91^ ^]^ Copyright 2020, Wiley‐VCH.

More recently, this technology has been extended to an adaptable synthetic PNPs fabrication method based on reactive electrojetting (**Figure** **4**A).^[^ ^91^, ^92^, ^94^, ^148^, ^149^, ^150^ ^]^ The jetting solutions include a dilute solution of proteins and reactive macromers, which will react together during and after the electrospraying process to stabilize the fabricated PNPs.^[^ ^91^ ^]^ Comparing the secondary structure of proteins in the PNPs to secondary structure of the native component proteins by CD spectroscopy confirmed that incorporation of the albumin proteins in the nanoparticles by EHD jetting process did not alter that secondary structure of albumin.^[^ ^149^ ^]^

Different reactive macromers were used to stabilize the EHD jetted PNPs (Figure 4B). Homobifunctional *N*‐hydroxysuccinimide (NHS)‐ester functionalized polyethylene glycol (PEG) based macromers were incorporated into the jetting solution with the protein.^[^ ^91^ ^]^ Specifically, 2 kDa *O*,*O*′‐bis[2‐(*N*‐succinimidyl‐succinylamino)ethyl]polyethylene glycol (PEG‐NHS) and 4,7,10,13,16,19,22,25,32,35,38,41,44,47,50,53‐hexadecaoxa‐28,29‐dithiahexapentacontanedioic acid *di*‐*N*‐succinimidyl ester (PEG‐NHS‐S) were used that reacted with the proteins’ amine groups.^[^ ^91^ ^]^ The reaction was completed after incubating the solidified PNPs at 37 °C for seven days.^[^ ^91^ ^]^ In another approach, after EHD jetting the protein solution with no macromer, solidified PNPs were exposed to glutaraldehyde vapor for 30 min to crosslink.^[^ ^91^ ^]^ Lastly, the intermolecular disulfide bonds of the protein were used to crosslink the PNPs.^[^ ^91^ ^]^ In this macromer‐free method, prior to EHD jetting, the protein was treated with trifluoroethanol and *β*‐mercaptoethanol to disrupt the protein structure and break native disulfide bonds within the protein.^[^ ^91^ ^]^ During the EHD jetting process, the evaporation of the solvents enabled the reformation of the disulfide bonds within the proteins resulting in the fabrication of stable PNPs on the counter electrode.^[^ ^91^ ^]^ These methods produced PNPs within the size range of 220–263 nm, being stable for 60 days at 4 °C.^[^ ^91^ ^]^ The PEG‐NHS‐S and macromer‐free crosslinking methods incorporated disulfide bonds within the PNPs enabling the particles to be redox‐responsive and break apart in the cellular reducing environments.^[^ ^91^ ^]^ This aspect of PNPs was confirmed where the uptake of transferrin nanoparticles crosslinked with different macromers in HeLa cells was studied.^[^ ^91^ ^]^ The PNPs that possessed disulfide bonds within their structure via their crosslinking strategy appeared more diffuse than the PEG‐NHS and glutaraldehyde crosslinked PNPs that remained punctate.^[^ ^91^ ^]^ To evaluate the feasibility of applying EHD jetting to different proteins, a library of proteins including human transferrin, insulin, hemoglobin, and lysozyme was explored (Figure 4C–F).^[^ ^91^ ^]^ The SEM image analysis showed a narrow particle distribution in the range of 68–99 nm.^[^ ^91^ ^]^ The hydrated diameter of particles measured by DLS was in the range of 223–269 nm (Figure 4G).^[^ ^91^ ^]^ The EHD co‐jetting process was also employed to fabricate bicompartmental PNPs.^[^ ^91^ ^]^ One compartment contained fluorescently labeled BSA and the other fluorescent human transferrin.^[^ ^91^ ^]^ The compartmentalization of PNPs was proved by structured illumination microscopy, demonstrating the applicability of this technology to synthetic PNPs (Figure 4H,I).^[^ ^91^ ^]^ These studies exhibited demonstrate the versatility, modularity, and adaptability of the EHD jetting process using various proteins and macromers.

One potential consideration related to the appropriate applications of electrospraying is the limited throughput of the system. The reliance on parallel capillaries to produce a multifluidic interface contributes to the low particle fabrication yield of this technique; therefore, a needleless EHD co‐jetting setup was designed to scale this process.^[^ ^93^ ^]^ In this platform a stable extended fluid interface was achieved by using a designed plate, where two different fluids were pumped to flow on each side of the plate and combine at the edge (the outlet of a microchannel). By applying a high electric field, multiple distinct Taylor cones were formed spontaneously along the fluid interface at the device outlet, resulting in about fivefold higher production rate of bicompartmental PNPs.^[^ ^93^ ^]^

Electrospraying is a modular atomization technique capable of producing micro‐ and nanoparticles for applications spanning from food to drug delivery. This technique has several merits including the retainment of biological activity and structure of proteins after fabrication, the tunability of process parameters, capability to load a myriad of therapeutic agents, and the ability to produce compartmentalized nanoparticles leading to more tunable features. However, low throughput is an underlying demerit encountered in electrospraying (Table 4). Efforts are currently being made to scale the process by adopting a needless electrospraying technique.

## 6 PNPs via Self‐Assembly

Unlike the aforementioned fabrication methods which form discrete PNPs with limited or no ordered structure, self‐assembly of proteins can result in precise and highly conserved protein clusters. Although the innate interactions are difficult to be specified individually due to their intrinsic complexity, they act highly site‐specific and orientational dependent, causing the self‐assembled nanoparticles to be ordered and monodisperse. Since this self‐assembly is governed by thermodynamics, engineering the sequences and structures of proteins hinges upon our ability to identify free energy minima, which has been extremely challenging and heavily relies on further progress of computational processes.^[^ ^33^, ^96^, ^97^ ^]^

### 6.1 Nanoparticle Preparation via Self‐Assembly Using Biologically Derived Proteins

**Figure 5:**
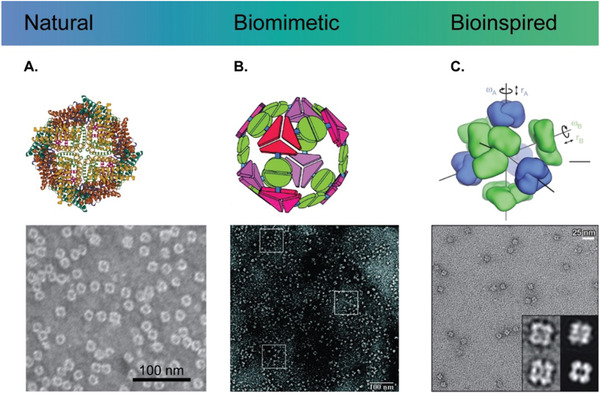
Structural illustration of self‐assembled protein nanoparticles. A) A natural ferritin nanoparticle (PDB: 3E6R) and a corresponding transmission electron microscope (TEM) image (scale bar: 100 nm). Reproduced with permission.^[^ ^157^ ^]^ Copyright 2017, National Academy of Sciences, USA. B) A biomimetic nanoparticle assembled from fusion proteins of natural trimeric and dimeric proteins and a TEM image of tetrahedral nanoparticles. Reproduced with permission.^[^ ^151^ ^]^ Copyright 2001, National Academy of Sciences, USA. C) A bioinspired nanoparticle assembled from de novo designed proteins and a corresponding TEM image. Each subunit has translational (*r*) and rotational (*ω*) degrees of freedom. Reproduced with permission.^[^ ^100^ ^]^ Copyright 2014, Springer Nature.

The initial self‐assembly approaches thus heavily relied on self‐assembling motifs from nature. In nature, many proteins undergo spontaneous association into PNPs, such as virus, ferritin, and encapsulin. Each nanoparticle is made up with natural proteins through inherent protein‐protein interactions. These natural PNPs are considered as initial candidates for therapeutic carriers due to their biocompatibility and biodegradability. In a set of studies, researchers have generated biomimetic self‐assembled nanoparticles.^[^ ^151^, ^152^, ^153^ ^]^ In the biomimetic PNPs,^[^ ^154^ ^]^ di‐ and trimeric assembly domains were incorporated into a fusion protein. With the parallel development of de novo protein design methods, a broader spectrum of bioinspired self‐assembled PNPs were achieved. In some cases, these approach resulted in non‐natural protein interactions and created properties (e.g., hyperstable constrained peptide) that went far beyond what Nature can offer.^[^ ^154^, ^155^, ^156^ ^]^ A synthetic protein with a sequence unrelated to natural proteins was designed to possess specific interaction domains having a minimized interfacial energy state obtained from computational calculations. Examples of natural, biomimetic, and bioinspired self‐assembled PNPs are illustrated in **Figure** **5**.

Viruses are a perfect example of self‐assembled PNPs in nature. Despite their inherent immunogenicity, high transfection efficacy makes virus (or virus‐like particle, VLP) a strong candidate for therapeutic carriers. Depending on the capsid protein assembly, the virions are largely classified into rod‐like, icosahedral, or complex shape. The icosahedral virions (diameter 20–800 nm) are finely defined monodisperse structures, comprising 60 of icosahedral asymmetric unit (IAU).^[^ ^158^ ^]^ If the IAU is made of single‐type capsid protein with a unique conformation, the triangulation number (T) is defined to 1. One example is the minute virus of mice (MVM, diameter ≈ 28 nm).^[^ ^159^ ^]^ When the IAU is composed of single‐type capsid protein with three different conformations (*T* = 3), the resulting icosahedral capsid would be composed of 180 subunits (*T* × 60), explained by quasi‐equivalence theory of Caspar and Klug.^[^ ^160^ ^]^ Examples include flock house virus (FHV, diameter ≈ 30 nm), Pariacoto virus (PaV, diameter ≈ 30 nm), brome mosaic virus (BMV, diameter ≈ 28 nm), cowpea chlorotic mottle virus (CCMV, diameter ≈ 28 nm), and tomato bushy stunt virus (TMSV, diameter ≈ 33 nm). The diameter of virion generally increases with increasing the triangulation number, *T*.^[^ ^161^ ^]^ For example, polyoma virus (diameter ≈ 40 nm) and simian virus 40 (SV40, diameter ≈ 45 nm) have *T* = 7 and bluetongue virus (diameter ≈ 69 nm) and rice dwarf virus (diameter ≈ 73 nm) have *T* = 13. If the IAU is formed by two or more types of capsid proteins, pseudo‐triangulation number is used. The cowpea mosaic virus (CPMV, *T* = p3, diameter ≈ 27 nm) comprises 60 IAUs which is containing different types of capsid proteins. For encapsulating cargo materials, VLP can be disassembled and reassembled depending on environments (pH and ionic strength).^[^ ^162^ ^]^ In addition, the capsid proteins can be genetically and chemically modified.^[^ ^163^, ^164^, ^165^, ^166^ ^]^ For example, DOX, an anti‐cancer drug, was covalently conjugated to the external surface of the CPMV through chemical modifications.^[^ ^167^ ^]^ CPMV was selected as a drug carrier for noninfectious toward mammals. Either stable amide bond or liable disulfide bond linkage between CPMV and DOX was inserted, resulting in aqueous suspension of DOX‐modified CPMV. From cytotoxic experiment with HeLa cells, both DOX conjugated CPMV showed effective cell killing. Cell viability treated with CPMV‐SS‐DOX displayed the same trend than a control group comprised of free DOX. These results suggest that disulfide bonds were broken in the culture media. In contrast, the amide bond conjugation exhibited higher stability, leading to the more effective cytotoxic agent at low DOX concentration regime. Also, empty CPMV can be employed as a cancer immunotherapy agent for in situ vaccination suppressing metastatic cancer in mouse model experiment.^[^ ^168^ ^]^

Recently, Steinmetz and coworkers reported a delivery of CCMV encapsulating oligodeoxynucleotides (ODNs) to improve antitumor efficacy.^[^ ^169^ ^]^ ODNs containing CpG have immunostimulatory effects, utilized in the treatment of allergy, infectious diseases, and cancer.^[^ ^170^, ^171^, ^172^ ^]^ According to the authors, there are two main reasons for using carriers instead of using free ODNs: i) protecting ODNs from nucleases in vivo and ii) improving the interactions with immune cells by suppressing their strong negative charges. The encapsulation was confirmed by UV absorbance (A260/A280) and native agarose gel electrophoresis. CCMV encapsulating ODN (CCMV‐ODN) showed a comparable structure to wild‐type CCMV particles, but possessed higher stability in physiological conditions (PBS, pH 7.4). Importantly, the encapsulated ODN was successfully protected from the DNase, confirmed by size‐exclusion chromatography (SEC). In addition, the CCMV‐ODN demonstrated improved cellular uptake by tumor‐associated macrophages compared to free ODN. Results of antitumor activity in vitro and in vivo exhibited that CCMV‐ODN had a higher therapeutic efficacy than free ODN.

As another example of natural PNPs, a ferritin complex (FTn) features a hollow spherical shell (diameter ≈ 12 nm) associated with 24 polypeptide subunits (Figure 5A). The natural ferritin is designed for storing and transferring iron ions in the form of FeOOH.^[^ ^173^ ^]^ With an internal diameter of 7 nm, finite magnetic iron oxide particles of 6 nm were synthesized inside apoferritin particles.^[^ ^174^ ^]^ The ferritin complexes can penetrate through nanoporous tissue barriers such as interstitial tissues or poorly permeable tumors since their diameter is small enough. In addition, ferritin has an intrinsic function to selectively bind to the transferrin receptor 1 (TfR1) which is highly expressed on rapidly dividing tumor cells.^[^ ^175^ ^]^ Huang et al. demonstrated that optimized PEGylated ferritin nanocages can deliver DOX to lung tumor tissue through airway mucus in which adhesive mucus gel protects most inhaled particulate matters.^[^ ^157^ ^]^ The key idea in here was tuning and optimizing surface density and length of PEG chains to avoid non‐specific adhesion by mucus gel. To achieve that, a mixed batch of PEGylated FTn and intact FTn in a certain ratio was subsequently treated to pH 2 and 7.4 for disassembly and reassembly, respectively.^[^ ^176^ ^]^ The resulting hybrid FTn (FTn/FTn‐PEG2k) was conjugated with DOX via an acid‐labile covalent bond. Instead of encapsulating drug, this covalent conjugation could avoid drug release before cellular uptake. Also, the acid‐labile bond allows DOX to be effectively released at endosomal acidic pH. As another example, Brenner and coworkers showed that supramolecular organization of proteins determined favored uptake in pulmonary marginated neutrophils during acute inflammation.^[^ ^149^ ^]^ Nanoparticles with agglutinated proteins (NAPs), including lysozyme‐dextran nanogels and albumin nanoparticles, were specifically accumulated in inflamed mice lungs even though the NAPs have different sizes (75–350 nm), shapes, zeta potentials, and protein components. In contrast to NAPs, ordered protein nanoparticles such as adenovirus, adeno‐associated virus, and horse spleen ferritin nanoparticles exhibited no specificity for the neutrophils in injured lungs. The authors speculated that the marginated neutrophils effectively recognize less patterned protein arrangements.

Encapsulins, found in bacteria and archaea, have gained increasing interests since their first discovery in 1994.^[^ ^177^ ^]^ The structures of encapsulin nanocompartments are icosahedron, consisting of protomers which are subunits similar to the capsid protein in viruses. When the triangulation number is 1, 60 of protomers assembles into 20–24 nm encapsulin PNPs;^[^ ^178^, ^179^, ^180^ ^]^ if T is 3 or 4, the diameters of encapsulin are 30–32 or 42 nm, respectively.^[^ ^181^, ^182^, ^183^ ^]^ Noticeably, pores of 5 or 7.3 Å diameters are present on the interfaces of the subunits, which are potentially useful as transporting channels of ions or small molecules.^[^ ^178^, ^182^ ^]^ Unlike virus particles which contains nucleic acids, natural encapsulin PNPs can encapsulate cargo protein such as ferritin‐like protein or dye decolorizing peroxidases.^[^ ^184^ ^]^ An encapsulin core operon typically has separate genes for the cargo protein and the protomers.^[^ ^178^ ^]^ The cargo protein can contain shorter peptide residues at their C‐terminus, called cargo‐loading peptide (CLP) which specifically binds to the protomer. This CLP helps the cargo proteins to be effectively encapsulated at interior sides of encapsulin PNPs. Moon et al. developed a targeted anticancer delivering nanocarrier from encapsulins.^[^ ^185^ ^]^ SP94‐peptide, hepatocellular carcinoma binding peptide, was either chemically or genetically conjugated to the exterior of encapsulin nanocompartments. Also, the prodrug aldoxorubicin (AlDox) was conjugated to a cysteine residue of encapsuling PNPs through chemical conjugation. This strategy required recombinant engineering of the base protein to introduce cysteine residues for the subsequent chemical modification, and hence, had no interference with the self‐assembly process itself. In another study, gold nanoparticle (AuNP) were encapsulated in PNPs.^[^ ^186^ ^]^ The interior surface of encapsulin has a specific binding affinity for CLPs. When the AuNP were decorated with these CLPs, protomers specifically bound to the CLP‐decorated AuNP. This specific binding affinity was strongly maintained even under high salt concentrations that result in screening of electrostatic interactions.

### 6.2 Nanoparticle Preparation via Self‐Assembly Using Biomimetic Proteins

Like natural proteins, self‐assembly of synthetic fusion proteins is also typically accomplished through the protein–protein interactions. Yeates and co‐workers created a genetically modified fusion protein comprised of trimeric bromoperoxidase, dimeric influenza virus M1 matrix protein, and rigid linker between them (Figure 5B).^[^ ^151^ ^]^ By imitating both trimeric and dimeric interaction motifs, the fusion protein spontaneously assembled into nanoparticles.^[^ ^154^ ^]^ Both natural proteins showed self‐associations without interfering with each other, implying that non‐bonded, site‐specific protein‐protein interactions were retained in the genetically modified variants. Resulting self‐assembled architectures were varied depending on the geometry of the building block (i.e., the angle between trimer and dimer), despite of identical molecular composition. For example, tetrahedral, octahedral, and icosahedral nanocages were formed with the angle of 54.7°, 35.3°, and 20.9°, respectively. Furthermore, 1D helical filaments, 2D layers, and 3D crystals could be designed as well. In following works, the tetrahedral protein nanocage (diameter ≈ 16 nm) was explored in detail with additional mutations to achieve an idealized model.^[^ ^152^, ^153^ ^]^

### 6.3 De Novo Self‐Assembly of Protein Nanoparticles Based on Computational Approaches

Precise assembly of proteins has been developed with aid of computational designed approaches. For example, Baker and co‐workers have developed a de novo design of proteins exploiting non‐natural protein‐protein interactions (Figure 5C).^[^ ^187^ ^]^ Finding the thermodynamic free energy minimum allows specific backbone structure and its sequence of amino acids to be identified. The computational result was substantiated by experiments. Natural trimeric building blocks can become mutated to have additional interfacial domains that enable binding with themselves at a specific angle. The resulting octahedral and tetrahedral cages were properly visualized and characterized by TEM. The research was expanded to realize ordered nanocages comprising multi components.^[^ ^100^ ^]^ In a follow‐up work, this de novo designed protein nanocages were able to encapsulate their own RNA genome, and evolve biological functionality such as genome packaging, stability in blood, and in vivo circulation time.^[^ ^101^ ^]^ This advanced strategy manipulating protein sequences and structures imparts the designed proteins to attain novel functions (so‐called bioinspired)^[^ ^154^ ^]^ such as hyperstability or selective ion‐channel.^[^ ^98^, ^155^, ^156^, ^188^, ^189^ ^]^

In a further extension of this work, Woolfson and co‐workers synthesized self‐assembled protein cage with coiled‐coil peptide.^[^ ^190^ ^]^ Two engineered synthetic peptides were utilized as building blocks: homotrimer (CC‐Tri3) and heterodimer (CC‐Di‐AB) composed of acid (CC‐Di‐A) and base (CC‐Di‐B) sequences. The peptides were designed to covalently link between CC‐Tri3 and either CC‐Di‐A or CC‐Di‐B through disulfide bond, which results in forming trimeric hub A or hub B. Due to the heterodimer motif of (CC‐Di‐AB), hexagonal networks with ≈5 nm pores were spontaneously produced after mixing of hub A and hub B. The flexible hexagonal networks ultimately formed self‐assembled cage‐like particles (SAGEs, diameter ≈ 100 nm). The researchers have thoroughly characterized the structures and stability of the helical peptides and their assemblies by DLS, CD spectroscopy with increasing temperatures, atomic force microscopy (AFM), and SEM. In a following work, the surface charges of SAGEs were concisely controlled by modifying the residues at the N or C terminus of CC‐Tri3.^[^ ^191^ ^]^ It clearly shows that positively charged SAGEs are extremely more efficient for cellular uptake by HeLa cells than negatively charged SAGEs.

**Figure 6:**
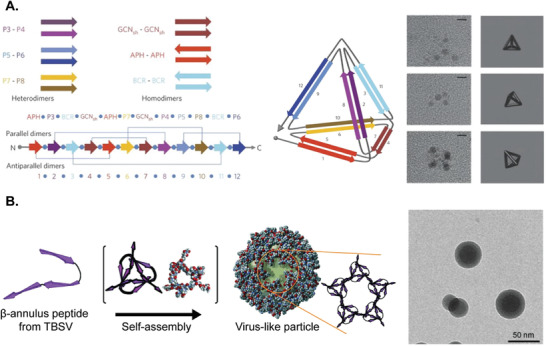
A) Schematic representation of self‐assembled polypeptide and corresponding TEM images (scale bar: 5 nm). Orthogonal peptide pairs (P3‐P4, P5‐P6, and P7‐P8) and homo‐dimeric peptide pairs (APH, BCR, and GCN_sh_) are connected in a polypeptide chain with a designed order. Numbers and arrows indicate the order and orientation of the coiled coil‐forming elements in the single polypeptide chain. Reproduced with permission.^[^ ^102^ ^]^ Copyright 2013, Springer Nature. B) Schematic illustration of virus‐like particles from synthetic 24‐mer *β*‐annulus peptides and a corresponding TEM image. Reproduced with permission.^[^ ^103^ ^]^ Copyright 2010, Wiley‐VCH.

Jerala and co‐workers used de novo design to engineer protein cages having different shapes (tetrahedron, four‐sided pyramid, and triangular prism) formed by coiled‐coil dimers (**Figure** **6**A).^[^ ^102^ ^]^ Inspired by DNA‐origami which is driven by highly specific pairwise attractions, coiled‐coil dimers can be employed to design protein‐origami cage.^[^ ^192^, ^193^ ^]^ The coiled‐coil dimers have similarities to the DNA duplex, which have elongated shape and pairwise complementarity. Specially, the coiled‐coil dimer is orientational specific: a parallel or an antiparallel orientation. A single polypeptide comprising orthogonally coiled‐coil forming sequences spontaneously self‐assembled into tetrahedral cages (diameter ≈ 6.9 nm), confirmed by DLS, AFM, and TEM, and small‐angle X‐ray scattering (SAXS).^[^ ^194^ ^]^ The topology of closed terminals was corroborated by bimolecular fluorescence complementation (BiFC). The shape of cages were not limited to tetrahedron but further explored to four‐sided pyramid or triangular prism by adopting 16 or 18 concatenated coiled‐coil forming segments.^[^ ^194^ ^]^ This self‐assembly strategy was still valid in vitro and in vivo. The mammalian cells were transfected by a plasmid encoding a protein designed to be assembled into the tetrahedron. The self‐assembled cages were localized in the cytosol of the HEK293 cells. For the in vivo test, the plasmid was introduced to the livers of mice. Correct folding was observed without any increase in inflammation or liver damage markers.

Du to its C_3_‐symmetric structure of virus and clathrin, synthetic trigonal *β*‐sheet forming peptide, Trigonal(FKFE)_2_, spontaneously assembled into spherical nanoparticles in acidic water (pH 3.3).^[^ ^195^ ^]^ Both CD spectroscopy and Fourier‐transform infrared spectroscopy confirms that antiparallel *β*‐sheets were formed. The diameter of nanoparticles was determined by AFM (≈35 nm), SEM (22–34 nm) as well as DLS (19 nm), which were well matched with expected size (16 nm for dodecahedron or 26 nm for icosahedron). The size was minimally affected by Trigonal(FKFE)_2_ concentration, implying the nanoparticles were discrete units. By adopting a similar strategy, Trigonal‐WTW was used as a building block for pH responsive, C_3_‐symmetric self‐assembled nanoparticles.^[^ ^196^ ^]^ These assemblies could be finite nanoparticles or nanofibers depending on the pH of the solution. When medium pH was lower than the polypeptide's isoelectric point (estimated PI ≈ 7.1), positive charges near core of Trigonal‐WTW electrostatically repelled each other, forming irregular, non‐defined structures. At pH 7, positive (near core) and negative (near periphery) charges in a Trigonal‐WTW helped the assemblies through antiparallel *β*‐sheets formation with aid of tryptophan zipper motif. At pH 11, existing negative charges only near the periphery enabled face‐to‐face assembly via hydrophobic stacking interaction at the core of Trigonal‐WTW. The researchers further developed the strategy to realize synthetic viral capsids from 24‐mer *β*‐annulus peptides, instead of using trigonal *β*‐sheet forming peptide (Figure 6B).^[^ ^103^ ^]^ The diameter was measured by TEM (≈44 nm), DLS (≈48 nm), and SAXS (≈50 nm). SAXS analysis was used to decipher its hollow structure with a wall thickness of ≈7 nm. From zeta‐potential characterization, *C*‐terminus and *N*‐terminus is toward exterior and interior, respectively.^[^ ^197^ ^]^ This synthetic viral capsid has been modified through various encapsulations and conjugation strategies involving polymers, peptides, gold nanoparticles, or even single‐strand DNA.^[^ ^198^, ^199^ ^]^

In summary, PNPs can be spontaneously fabricated via self‐assembly of natural, modified, or de novo proteins. The underlying principle is based on complex protein‐protein interactions which can be distinguished from other fabrication methods. Resulting PNPs are highly conserved protein clusters, behaving site‐specific, orientational dependent, ordered, and monodisperse. However, the system strongly relies on computational work with atomic‐scale accuracy. Another concern relates to the potential immunogenicity of self‐assembled PNPs since the building block proteins are mostly mutated or de novo designed except for natural PNPs (Table 4). Also, stability of self‐assembled PNPs in blood stream is a major concerns as complicated intermolecular interactions may interfere the self‐assembly remains an unanswered question. Despite of these shortfalls, the molecular pecision and unlimited protein design space make this approach unique and promising for PNP development in the context of nanoparticle‐based delivery system.

## 7 Strategies for Stabilizing PNPs

Owing to protein's structural flexibility and complexity, they can spontaneously undergo refolding and aggregation depending on temperature, pH, salt type, salt concentration, and protein concentration.^[^ ^200^ ^]^ Even in physiological environments, a native protein is only marginally stable and can assume a range of structural conformations. Partially unfolded proteins can interact with each other or other proteins, potentially resulting in aggregated structures. Alternatively, previously assembled protein nanoparticles can be disassembled releasing their therapeutic payload in the process. To harness the vast potential benefits for PNPs, effective stabilization becomes a prerequisite.

**Figure 7:**
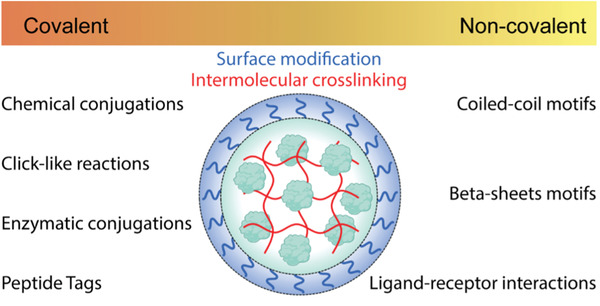
Strategies of stabilization for PNPs. Created with BioRender.com.

General strategies for PNP stabilization include either intermolecular crosslinking or surface modification via covalent or non‐covalent bonding (**Figure** **7**). Intermolecular crosslinking can be realized either via incorporation of synthetic crosslinkers, which then become a part of the final PNP composition, or via activation of intrinsic reactive groups using external stimuli such as heat,^[^ ^201^ ^]^ light,^[^ ^202^, ^203^, ^204^, ^205^ ^]^ pH,^[^ ^157^, ^176^ ^]^ or reducing agents.^[^ ^117^ ^]^ (i.e., self‐crosslinking).^[^ ^206^, ^207^ ^]^ For example, Wang et al. initially broke native intramolecular disulfide bonds in HSA with glutathione as a reducing agent, followed by formation of nanoparticles via the desolvation technique.^[^ ^117^ ^]^ After dialysis to remove the reducing agent, HSA nanoparticles remain stable in different pH solutions as well as fetal bovine serum solution. Only few accessible cysteine residues in proteins may limit the possibility of intermolecular crosslinking.^[^ ^208^, ^209^ ^]^ This potential problem can be mitigated by introducing additional‐SH functional groups from a reaction of the Traut's reagent with lysine residues.^[^ ^210^ ^]^

Stabilization through surface modification is noticeably different from intermolecular crosslinking in that non‐specific aggregation is effectively avoided by decorating the nanoparticle surface with hydrophilic moieties. PEGylation is the representative approach in this category. Kim et al. conjugated PEG to the elastin‐like proteins (ELPs) through amide bond formation.^[^ ^201^ ^]^ In contrast to non‐PEGylated ELPs, which showed non‐specific aggregation at elevated temperature, the PEGylated ELPs displayed a relatively uniform size of nanoparticles.

Bonding strength is an important consideration for the stabilization. Appropriate chemical bonds or physical bonds should be selected depending on intended applications. For example, cell killing efficacy of DOX‐conjugated CPMV via disulfide bond (CPMV‐SS‐DOX) had comparable results to free DOX in a HeLa cell viability test, suggesting that the disulfide bonds were unintentionally cleaved in the cell culture medium.^[^ ^167^ ^]^ The CPMV‐DOX conjugated via amide bond displayed exceptionally stability without noticeable bond cleavage. On the other hand, considering the necessity of nanoparticle degradation at an intended place, stabilization with non‐covalent bonds can be preferred.^[^ ^105^ ^]^ The approaches of both covalent and non‐covalent bonding stabilizations will be described with few examples below. Also, the reader is referred to excellent reviews regarding advanced conjugation strategies.^[^ ^211^, ^212^, ^213^, ^214^, ^215^, ^216^, ^217^ ^]^

### 7.1 Covalent Bonding Stabilization

In covalent bonding stabilization (CBS), a new covalent bond is formed between molecules of interests by sharing electron pairs through a chemical reaction. Covalent bonds are one of the strongest chemical bonds in nature; the bond dissociation energies for most organic molecules are in a range of 100–1000 kJ mol^−1^ at 298 K.^[^ ^218^ ^]^ Unless additional dissociative reaction occurs (e.g., hydrolysis, disulfide reduction), newly formed covalent bonds are extremely stable and usually irreversible.

Considering proteins dispersed in aqueous medium, hydrophilic polar residues are exposed to the exterior and hydrophobic non‐polar residues are buried towards the interior of the protein. Thus, polar residues are easily accessible and highly reactive compared to their nonpolar counterparts. Among polar residues, lysine is usually the first consideration for chemical conjugations (**Table** **7**). Lysine is abundant on the surface of many proteins and the *ε*‐amino groups enable ease of accessibility to conjugation reagents.^[^ ^208^ ^]^ The *ε*‐amino groups (pKa ≈ 10.5) are deprotonated and act as nucleophiles in basic condition. As an activated ester, *N*‐hydroxy succinimide (NHS) esters readily react with lysines to form stable amide bonds around neutral or slightly basic pH solution (pH 7–8). Amine‐containing buffers should thus be avoided for amine‐selective reaction. Although NHS‐ester can easily be hydrolyzed in aqueous solution and has low coupling efficiency, it is one of the most widely used conjugations due to its biocompatibility and chemoselectivity.^[^ ^219^, ^220^ ^]^ The resulting aliphatic amide bonds are exceptionally stable as well.^[^ ^221^ ^]^ As an example, 3,3’‐dithiobis(sulfosuccinimidyl propionate) (DTSSP) was used for crosslinking CCMV capsid proteins.^[^ ^222^ ^]^ Unlike wild‐type CCMV particles which can disassemble at pH 7.5, the crosslinked CCMV exhibited extended stability at physiological conditions at 37 °C without any disruptions of particle structure and functionality of cargos. Additionally, incorporated disulfide bond in the DTSSP imparted redox‐responsive reversibility to the CCMV carrier.

**Table 7:** Common chemical conjugation of amino acids. Created with BioRender.com

Amino acid	Residue	Reactant	Product	Note
Lysine	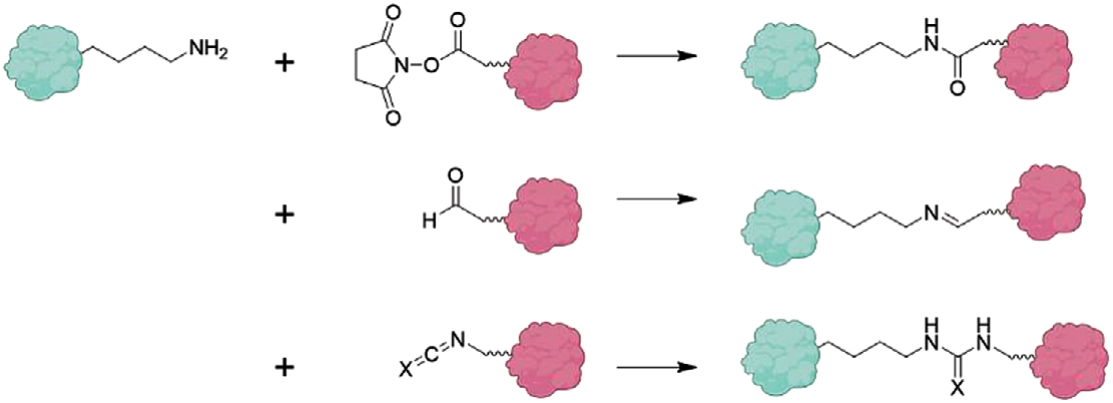			X = O or S
Cysteine	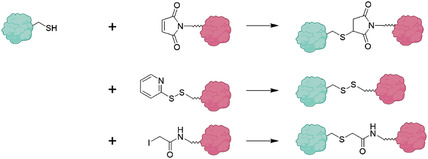			
Glutamic acid or Aspartic acid	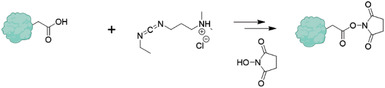			

Upon reaction with the *ε*‐amino group, aldehydes reversibly form imine bonds which can be further reduced to stable, irreversible secondary amine. Sodium cyanoborohydride, a mild reducing agent, helps with the selective reduction of imines and is generally considered superior to sodium borohydride which can reduce aldehyde to alcohol, leading to a low coupling efficiency.^[^ ^223^ ^]^ Direct crosslinking of PNPs can also be achieved by reaction with glutaraldehyde in vapor phase.^[^ ^91^ ^]^ Although the vapor phase crosslinking is common for fibers, crosslinking PNPs in vapor phase is rarely reported.^[^ ^224^, ^225^, ^226^, ^227^, ^228^ ^]^ Vapor phase crosslinking specially has a benefit by avoiding disassembly of uncrosslinked PNPs in solution phase. PNPs obtained from EHD jetting were placed in an air‐tight container with a certain amount of glutaraldehyde solution. Because the PNPs are directly exposed to gaseous phase, vaporized glutaraldehyde can react to lysine residues on the surface of PNPs.

Isocyanates or isothiocyanates undergo a reaction with amines to form stable urea or thiourea groups, respectively. Due to the low stability of isocyanate, isothiocyanate is preferred in bioconjugation. Fluorescein isothiocyanate (FITC) is one of popular examples for this conjugation. Analogous to NHS‐esters, the isothiocyanate can also have reversible reactions with other nucleophilic alcohol or thiol containing amino acids such as cysteine, serine, threonine, or tyrosine.^[^ ^211^ ^]^

Despite the low abundance of cysteine residues, they are popular for bioconjugation or crosslinking because only a small fraction of the cysteines is free and accessible. For example, only one of the cysteines (cys34) in HSA is unpaired.^[^ ^229^ ^]^ Sulfhydryl and thiolate groups are one of the most nucleophiles among standard amino acids, leveraging versatile conjugating reactions. Iodoacetamide groups undergo irreversible alkylation with sulfhydryl group at alkaline conditions (pH 7.5–9). Adjusting the pH or exchanging the halogens allows for selective cysteine conjugation although the iodoacetamide is able to react with other nucleophiles.^[^ ^,230^ ^]^ Maleimide reacts with the cysteine with fast kinetics and high selectivity around neutral conditions (pH 6.5–7.5). In basic environments, side‐reactions with lysine or hydrolysis can occur, resulting in low coupling efficiency. The cysteines are involved in protein folding by formation of disulfide bonds, which can be reversibly reduced and oxidized. To reduce the disulfide bonds, reducing agents such as dithiothreitol (DTT), glutathione (GSH), or tris(2‐carboxyethyl)phosphine (TCEP) are added into a protein solution prior to the thiol conjugations. The reversible disulfide bonds can be applicable in disulfide exchange reactions which involves breaking the existent S—S bonds and forming new S—S bonds with the new desired biomolecules conjugated. For example, 2‐pyridyl disulfide has a leaving group to shift the reaction equilibrium.^[^ ^231^ ^]^ Since the reduction of disulfide bonds may alter the entire protein structure, stability, and functionality, it is critical to retain the disulfide bridges after conjugations. Development of *α*,*β*‐unsaturated bis‐thiol alkylating reagents or dibromo‐maleimide successfully resolved the problem.^[^ ^232^, ^233^, ^234^ ^]^

Carboxylic acid groups in glutamic and aspartic acid are often converted into a more reactive ester by reacting with water‐soluble carbodiimides such as 1‐ethyl‐3‐(3‐dimethylaminopropyl)carbodiimide (EDC). Slightly acidic conditions are necessary to protonate the carboxylic acid for the reaction with EDC (pH > 4.5). Once the EDC is conjugated to the carboxylic acid, the resulting *O*‐acylisourea group readily reacts toward amine to form amide bond at slightly basic conditions (pH 7–8).^[^ ^206^ ^]^ Addition of NHS to the *O*‐acylisourea group forms relatively stable NHS ester, leading improved efficiency of amide formation.^[^ ^235^ ^]^

Azide groups have frequently been used for either the Staudinger reaction or azide‐alkyne cycloaddition. Triarylphosphine selectively reacts with the azide moiety to produce an intermediate iminophosphorane which is further hydrolyzed to eventually form the amide bond.^[^ ^236^ ^]^ Non‐natural amino acids containing azide (e.g., azidohomoalanine, AHA) in recombinant proteins were chemo‐selectively coupled to triarylphosphine in crude cell lysates.^[^ ^237^ ^]^ In addition, this conjugation has directly been utilized in crosslinking alginate or synthetic methacrylic polymers.^[^ ^238^, ^239^, ^240^ ^]^ Copper(I)‐catalyzed azide‐alkyne cycloaddition (CuAAC), as one type of click reactions, is rapid, biocompatible, chemo‐selective, and high yield reaction at ambient condition with insensitivity to oxygen and water. Despite a well‐established, convenient tool, the requirement of using Cu(I) catalyst greatly limits the usage in a cellular system because of its cytotoxicity. In efforts to resolve this, electron‐deficient alkyne was designed by Li et al.^[^ ^241^ ^]^ Although the electron‐deficient alkyne can undergo Michael reaction, it is remarkably efficient and practical for various field of researches.^[^ ^241^, ^242^, ^243^, ^244^ ^]^ Simultaneously, Bertozzi et al. and co‐workers has developed strain‐promoted azide‐alkyne cycloaddition (SPAAC).^[^ ^245^ ^]^ Strained cyclooctyne derivates have been extensively utilized into in vivo intracellular visualization, maximizing copper‐free feature.^[^ ^246^, ^247^, ^248^ ^]^

Significant efforts have been devoted to understanding and improving enzymatic conjugations. As one of the major benefits, enzymes catalyze conjugating reactions at mild conditions; most enzymatic reaction occurs at neutral pH and moderate temperature in the absence of an organic solvent. Enzymatic reactions can occur in complex biological environments without undesirable effects on other biomolecules. In addition, analogues to the click reactions, enzymatic conjugations have fast kinetics, site‐specificity, and high yield without unwanted byproducts, and form stable covalent bonds. Unlike click reactions, the enzymatic conjugation does not require non‐natural amino acids but rather involves standard amino acids. For example, transglutaminase (TG) forms an isopeptide bond by reacting the *γ*‐carboxamide group in glutamine with the *ε*‐amino group in lysin. Fuchs et al. reported that desolvated gelatin nanoparticles were crosslinked by TG.^[^ ^249^ ^]^ Various parameters, including temperature, pH, buffers, and reaction time were evaluated for the effective crosslinking. According to the authors, preferred crosslinking conditions are identified as ion‐free water medium at a neutral pH for 12 h at 25 °C. As another work, electrostatic complexes of dextran‐grafted whey protein isolates (WPI) and chondroitin sulfate (Chs) were stabilized by TG.^[^ ^250^ ^]^ The crosslinked complexes exhibited exceptional stability over a wide range of pH (1–10) and at high ionic strength. The complexed nanoparticles remain stable even after heating at 90 °C for 30 min. In vitro gastrointestinal release test displayed that the TG crosslinked nanoparticles featured controlled releasing cinnamaldehyde which is a model bioactive compound.

Lysyl oxidase (LO) and plasma amine oxidase (PAO) convert the *ε*‐amino group into reactive aldehyde which can consecutively react to another *ε*‐amino group to form reversible imine group. Bakota et al. demonstrated lysine‐containing peptide hydrogels which is self‐crosslinked by LO and PAO in standard mammalian cell culture conditions (with fetal bovine serum), respectively.^[^ ^251^ ^]^ Since the serum in standard cell culture media already has LO, the peptide hydrogel was obtained without added LO. Addition of *β*‐aminopropionitrile (*β*APN) as an inhibitor of the enzyme clearly inhibited the self‐crosslinking.^[^ ^252^ ^]^ Although this example is relevant to the crosslinking hydrogels, the approach is not limited to the hydrogels and can be applied into stabilizing PNPs.

With hydrogen peroxides (H_2_O_2_), horseradish peroxidase (HRP) can oxidize hydroxyphenyl groups in tyrosine and form radicals which carry out crosslinking each other. Tyrosine‐tagged proteins were successfully crosslinked by HRP and H_2_O_2_ at 37 °C in Tris buffered solution (pH 8).^[^ ^253^ ^]^ The researchers observed that three‐consecutive tyrosine tagged protein (CY3) had lower crosslinking efficiency than single tyrosine tagged protein (CY1) which can be understood by two explanations; the bulky tyrosine residues may inhibit HRP recognition and the generated radicals have intrinsically higher chance to react tyrosine by next, resulting in intramolecular crosslinking.

Sortase A (SrtA) recognizes a specific sequence of amino acid (LPXTG) to catalyze reversible cleavages of amide bonds between threonine and glycine and formations of thioesters. The thioesters can further react with other nucleophiles. For example, LPXTGX and GGGG separately functionalized hyaluronan biopolymers are crosslinked with an existence of SrtA.^[^ ^254^ ^]^ Cytocompatibility was corroborated by encapsulating and culturing human chondroprogenitor cells for 12 days (viability > 90%). Chen et al. demonstrated multifunctional conjugations on the surface of E2 nanoparticles which is one of natural PNPs having a diameter of ≈24 nm.^[^ ^255^ ^]^ GGG sequence was genetically inserted to the *N*‐terminus of E2 subunit. After formation of E2 nanoparticles, LPETG‐tagged elastin‐like protein (ELP) and tetrameric LPETG‐tagged *β*‐galactosidase were sequentially added with SrtA. The modified E2 PNPs possessed thermo‐responsive property for the conjugated ELP and interparticle crosslinking structures due to the tetrameric *β*‐galactosidase.

Hexahistidine‐tags (His‐tags) have been utilized in protein purification, labeling, or immobilizations.^[^ ^256^, ^257^ ^]^ Electron‐donating groups in His‐tags make coordinate bonds to the metal ions such as Cu^2+^, Ni^2+^, Zn^2+^, or Co^2+^. The coordinate bonds are intrinsically reversible although they are sharing electrons so considered as covalent bonds. Minten et al. genetically incorporated the His‐tag to the CCMV capsid protein so the CCMV particles (His‐CCMV) were stabilized with Ni^2+^ at neutral pH where wild‐type CCMV can disassemble.^[^ ^258^, ^259^ ^]^ After addition of ethylenediaminetetraacetic acid (EDTA), the His‐CCMV particles were disappeared, indicating Ni^2+^ ions are essential for the stabilization.

Alternatively, SpyTag/SpyCatcher systems have been pursued as peptide‐protein pairing techniques, that have been quickly adopted in various research fields.^[^ ^260^, ^261^, ^262^, ^263^, ^264^ ^]^ The bio‐orthogonal reaction between an aspartic acid residue of the SpyTag (13 amino acids) and a lysine in SpyCatcher (113 amino acids) creates an irreversible link between two proteins. This reaction is extremely fast, autocatalytic without requirement of any enzymes or cofactors, and insensitive to temperatures, redox conditions, buffers, and pH. Effective conversion has been observed under in vivo, and in vitro conditions. Lee et al. recently reported human epidermal growth factor receptor 2 (HER2)‐targeting PNPs based on the SpyTag/SpyCatcher system.^[^ ^265^ ^]^ SpyTag was conjugated to HSA molecules through two‐step coupling reactions; lysine residues in HSA reacted to *N*‐succinimidyl 3‐maleimidopropionate (NHS‐MAL). The maleimide‐decorated HSA further reacted to SpyTag‐cysteine. Then, the modified HSA was incubated with glutathione (GSH) to reduce innate disulfide bonds, followed by desolvating with ethanol. Resulting desolvated‐PNPs (diameter ≈ 150–180 nm)were self‐crosslinked via newly formed disulfide bonds and possessed surface‐accessible SpyTag. SpyCatcher fused HER2 affibody molecules (SC‐HER2Afbs) were successfully conjugated to the PNPs, forming specifically HER2‐targeting PNPs. The PNPs selectively accumulated in tumors expressing HER2 and exhibited distinguishable anticancer inhibitory effect when photothermal agent was encapsulated in the PNPs.

### 7.2 Non‐Covalent Bonding Stabilization

In non‐covalent bonding stabilizations, multiple weaker interactions are employed to maintain the nanostructure of proteins. Although electrostatic interactions such as ion‐ion interaction, ion‐dipole interaction, hydrogen bonding interaction, and dipole‐dipole interaction have relatively lower bonding energies than the covalent bonding energy, cooperative electrostatic interactions and the hydrophobic interactions can stabilize the protein structures, including the secondary, tertiary, and quaternary structures.^[^ ^266^ ^]^ With nature as inspiration, researchers have discovered and engineered specific interaction motifs of peptides and proteins. Some established interaction motifs to stabilize protein nanostructures are discussed below.

**Figure 8:**
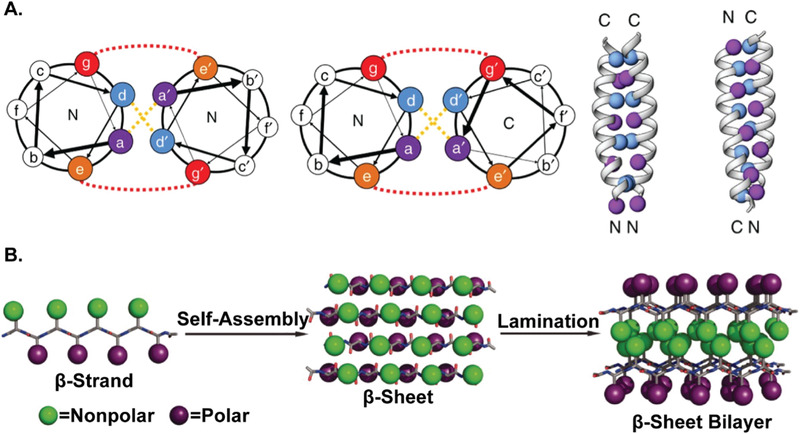
A) Graphical representation of coiled‐coil assembled structure. Yellow and red dashed lines indicate hydrophobic interactions and electrostatic interactions, respectively. Heptad repeats have pairwise attractions in a parallel or antiparallel orientation. Reproduced with permission.^[^ ^194^ ^]^ Copyright 2017, Springer Nature. B) Illustration of self‐assembly process from amphipathic *β*‐strands into *β*‐sheets bilayer. Reproduced with permission.^[^ ^267^ ^]^ Copyright 2012, Wiley Periodicals, LLC.

Francis Crick and Linus Pauling independently discovered the coiled‐coil (or super‐helix) structure by observing the tilted angle (≈18 °) of *α*‐helices in *α*‐keratin.^[^ ^268^, ^269^ ^]^ The two or more *α*‐helices can be associated through multiple intermolecular interactions with highly ordered structures (**Figure** **8**A). In most common cases, helices in coiled‐coil wrap around each other into a left‐handed helix with 3.5 residues for each complete turn, implying the basic structure of each helix has heptad repeats: (**a**–**b**–**c**–**d**–**e**–**f**–**g**)*_n_* in one helix, and (**a′**–**b′**–**c′**–**d′**–**e′**–**f′**–**g′**)*_n_* in the another for a case of dimerized coiled‐coil.^[^ ^270^, ^271^ ^]^ The **a**, **d** residues have nonpolar nature (e.g., Leu or Ala), forming hydrophobic cores at the interfaces of coiled‐coil. These hydrophobic cores primarily contribute overall stability of coiled‐coil by facilitating attraction between the helices in aqueous medium. The **e**, **g** residues possess charged groups (e.g., Lys or Glu) to control repulsive or attractive interaction between the helices. Importantly, the charged residues mostly determine the association specificity, indicating homo‐ or heterotypic pairing and parallel or antiparallel orientation.^[^ ^272^ ^]^ For example, strong attraction of **e**–**g′** results in parallel orientation. Similarly, strong attractions of **e**–**e′** and **g**–**g′** give rise to antiparallel orientation. The **b**, **c**, **f** residues mostly own polar, hydrophilic properties, providing aqueous solubility as well as hydrophobic interactions. More specifically, the **b** and **c** positions prefer uncharged residues which are less interfering coulomb interactions of **e** and **g** residues.^[^ ^273^ ^]^ The **f** residue can be charged amino acid for preventing aggregation.

Amphipathic peptides consisting of alternating nonpolar and polar amino acids can spontaneously assemble into *β*‐sheets and highly ordered fibrils depending on their compositions, length, and environments (Figure 8B). As one of the most clear studies, alternating poly(Phe‐Lys) were synthesized to form *β*‐sheets with different conditions such as pH and salt concentrations.^[^ ^274^ ^]^ The presence of *β*‐sheets was determined from CD spectra when ionic repulsions between Lys residues are diminished by increasing pH or ionic strength. It is important to note that structures of corresponding random poly(Phe‐Lys) displayed distinguishable CD spectra from that of alternating poly(Phe‐Lys) in the same environment, implying a critical role of amino acids sequence in self‐assemblies. Brack et al. depicted detailed conformations of alternating poly(Val‐Lys) in *β*‐sheets powders from X‐ray diffraction analysis.^[^ ^275^ ^]^ Postulating existence of *β*‐sheet bilayer, it can be rationalized that each surface of *β*‐sheet has prevalently hydrophobic Val and hydrophilic Lys, respectively, in agreement with staggered side chains having a specific range of dihedral angles.^[^ ^276^ ^]^ The *β*‐sheet is an assembly of extended oligopeptides in a specific orientation by intermolecular hydrogen bonding between amide bonds in backbone chains. Similarly, Schneider and co‐workers designed 20‐mer peptide (MAX1) containing (VK)_4_‐V^D^PPT‐(KV)_4_.^[^ ^277^ ^]^ Although the control peptides (VK)_4_ remained without self‐assembly in high pH, the MAX1 are reversibly self‐assembled into *β*‐sheets and fibrils in the basic condition. Due to the incorporation of the *β*‐turn‐forming tetrapeptide (V^D^PPT), the MAX1 is supposed to transform into the *β*‐hairpin structure prior to the formation of *β*‐sheets. This *β*‐hairpins facilitated the self‐assembly both laterally and facially by hydrogen bonding between hairpins and hydrophobic interaction between Val‐rich faces, respectively.

Similarly, electrostatic complementarity was leveraged to induce co‐assembly of anionic and cationic *β*‐sheet‐forming peptides.^[^ ^278^ ^]^ Each *β*‐sheet‐forming peptides cannot spontaneously associate without its counterpart due to the electrostatic repulsion. When only both oppositely charged peptides are co‐existing, the self‐assembled fibril structure could be observed. The co‐assembled peptides have an ordered structure but not disordered aggregation from charge‐charge complexes. Also, it is proven that the hydrophobic phenylalanine in appropriate sequence was required to form *β*‐sheets. Mutated peptides from phenylalanine to proline showed an absence of distinct supramolecular structures, implying the importance of hydrophobic interactions in self‐assembly.

The ligand‐receptor interactions representatively include avidin‐biotin, antibody‐antigen, hormone‐receptor, enzyme‐substrate, and lectins‐glycoprotein.^[^ ^279^ ^]^ Several non‐bonded attractions of the ligand surface cooperatively, reversibly act on a specific conformation of the receptor surface.^[^ ^280^, ^281^ ^]^ Binding affinity (dissociation constant, *K*_D_) of the interactions varies from 10^−3^ to 10^−15^ m, depending on the dominant non‐covalent interactions (e.g., ionic bonding, hydrogen bonding, or hydrophobic interaction) at the binding domains.^[^ ^266^, ^282^ ^]^ The avidin‐biotin interaction is known to the strongest binding affinity (≈10^−15^ m) among non‐bonded interactions.^[^ ^283^ ^]^ This exceptionally strong non‐covalent interaction may be explained by the cooperative hydrogen bonds and hydrophobic interaction at the binding pockets.^[^ ^284^ ^]^ Non‐covalent crosslinking has been accomplished by adopting these avidin‐biotin interactions. Norioka et al. reported gelation of biotinylated *tetra*‐poly(ethylene glycol) with avidin.^[^ ^285^ ^]^ Reversibility is clearly demonstrated by transition from gel‐state to sol‐state after an addition of free biotin into the system. Furthermore, owing to its site‐specificity, the avidin‐biotin interaction can be utilized for crosslinking of functional proteins without interfering bioactivity. Kamiya and co‐workers showed spontaneous formation of protein supramolecular complex (PSC) from biotinylated bacterial alkaline phosphate (AP) and streptavidin.^[^ ^286^, ^287^ ^]^ The length and the number of biotin groups, as well as biotinylated sites on dimeric AP contributed the structures of PSCs. Considering the two binding sites in a same face of streptavidin, assembly of bis‐biotinylated ligand with streptavidin results in one‐dimensional supramolecular structure.^[^ ^288^, ^289^ ^]^ As another interesting example, avidin nanoparticles were stabilized via PEGylation.^[^ ^290^ ^]^ The avidin nanoparticles (diameter ≈ 115 nm) were spontaneously formed by mixing avidin and plasmid DNA (4.7 kb) due to the high affinity of avidin for nucleic acids.^[^ ^291^ ^]^ Biotin‐PEG (5 kDa) was added to prevent non‐specific aggregation of the nanoparticles. Also, biotinylated immunoglobulin‐*G* (*b*‐IgG) or/and biotinylated horseradish peroxidase (*b*‐HRP) were also incorporated into the avidin nanoparticles for multi‐functionalization. Since this system is biocompatible and biodegradable, it can be applicable to in vitro and in vivo experiments such as targeted cancer therapy.^[^ ^292^, ^293^, ^294^ ^]^ Howarth and co‐workers developed genetically modified avidin subunits, leading the modified avidin to bind biotinylated ligands under precisely controlled *cis*‐ or *trans*‐arrangement.^[^ ^295^, ^296^ ^]^ Since the interactions are extremely sensitive to the interfacial conformations between the ligand and receptor, the binding affinity is also affected by selectivity and molecular orientation.^[^ ^297^, ^298^, ^299^ ^]^

Other than avidin‐biotin interactions, countless pairwise ligand‐receptor interaction can be leveraged for crosslinking or stabilizing structured materials. Arginine‐glycine‐aspartic acid (RGD) sequences can be recognized by integrin, heterodimeric transmembrane protein. Rheological characterizations suggest that the integrins on cells are dynamically crosslinked to the RGD‐containing alginates.^[^ ^281^ ^]^ Heme‐Hemeprotein interaction adopted for 1D supramolecular self‐assemblies.^[^ ^300^ ^]^ Cytochrome b_562_ (cyt b_562_) was mutated from His63 residue to Cys63 residue (opposite site of heme‐binding site), followed by conjugation with heme‐terminated short chain. PH‐dependent denaturization allows the system to have reversible self‐assembly. Dimensionality of supramolecules was successfully tuned by introducing heme triad, promising a fabrication of more complicated architectures by changing specific binding site, building block structure, and molar ratio.^[^ ^301^ ^]^

Lectins‐glycoprotein interaction, as another interaction motif, is applicable to crosslink carbohydrate‐containing molecules.^[^ ^302^, ^303^, ^304^, ^305^ ^]^ Brewer et al. have rigorously characterized and reported several lectin‐carbohydrate crosslinking systems.^[^ ^306^, ^307^, ^308^ ^]^ For example, a mixture of tetrameric concanavalin a (Con A) and tetrameric soybean agglutinin (SBA) which possesses a oligomanose‐type chain precipitated in aqueous solution, forming homogeneous crosslinked complexes.^[^ ^309^ ^]^ The structure of dynamic complexes depends on molar ratio and quaternary structures of Con A and SBA. This finding is further proved by expanding to other pairs of lectin‐glycoproteins, including Con A–Lotus tetragonolobus isolectin A (LTL‐A), Con A–Erythrina cristagalli lectin (ECL), asialofetuin (ASF)–ECL, and so on.^[^ ^310^, ^311^, ^312^ ^]^ Isothermal titration calorimetry (ITC) measurements revealed that binding enthalpy (∆*H*) of tetravalent ligand and monovalent ligand were −53.0 and −14.7 kcal mol^−1^, respectively, which suggests that the multivalent ∆*H* is approximately proportional to the number of bindings.^[^ ^313^ ^]^

One of special motifs for crosslinking protein nanostructures is photo‐induced protein association. Photo‐responsive recombinant protein UVR8‐1 can switch from homodimer to monomer upon UV irradiation.^[^ ^203^ ^]^ The crosslinked protein hydrogel rapidly changed into solution state after UV exposure for 20 min. The gel state was recovered after being ≈2 h at room temperature, proving the association and dissociation is totally reversible. Lyu et al. developed fully optically controlled crosslinking system by introducing a mutant photoswitchable fluorescent protein Dronpa145N, allowing associated tetramer under light *λ* ≈ 500 nm and dissociated monomer under light *λ* ≈ 400 nm.^[^ ^204^ ^]^ Also, a cell‐compatible, low light energy required crosslinking system was developed by Hörner et al.^[^ ^202^ ^]^ Cyanobacterial photoreceptor Cph1 reversibly changes to dimers under *λ* ≈ 660 nm and monomers under *λ* ≈ 740 nm. Although this photo‐induced protein association has not been utilized into stabilization for PNPs yet, this approach will allow PNPs to release their payloads at localized positions when only external light is applied.

**Table** **8** briefly summarizes and compares representative stabilization strategies.

**Table 8:** Comparison of stabilization strategies

Strategies	Example	Bond type	Prominence in research community	Selectivity	Requiring protein modification	Applicable pH	Biocompatibility	Stability of resulting bond	Protein structure preservation
Chemical conjugation	NHS ester	C	+++	++	No	7–9	+++	+++	++
	Aldehyde	C	+++	+	No	7–9	+	++	++
	Isothiocyanate	C	++	++	No	≈9	+	+++	+
	Iodoacetamide	C	+	++	No	7.5–9	+++	+++	+++
	Maleimide	C	+++	++	No	6.5–7.5	+++	++	+++
	Disulfide exchange	C	+++	+++	No	6–9	++	++	+
	EDC	C	+++	++	No	4–6	++	+++	++
Click‐like reactions	Staudinger reaction	C	+	+++	Yes	4–11	+++	+++	+++
	CuAAC	C	+++	+++	Yes	4–11	+	+++	+++
	SPAAC	C	+++	+++	Yes	4–11	+++	+++	+++
Enzymatic conjugation	Transglutaminase	C	++	++	No	≈7.4	+++	+++	+++
	Lysyl oxidase	C	+	++	No	≈7.4	+++	+++	+++
	Horseradish peroxidase	C	++	++	No	≈7.4	+++	+++	+++
	Sortase A	C	++	+++	Yes	≈7.4	+++	+++	+++
Peptide Tags	His‐tags	C	+++	+++	Yes	7.5–8	++	++	+++
	SpyTag/SpyCatcher	C	++	+++	Yes	5–8	+++	+++	+++
Coiled‐coil motifs	Coiled‐coil motifs	NC	++	++	Yes	5–8	+++	++	+++
Beta‐sheets motifs	Beta‐sheets motifs	NC	+	+	Yes	4–6.5	+++	++	++
Ligand‐receptor interactions	Avidin‐biotin	NC	+++	++	Yes	2–11	+++	+++	+++
	Antibody‐antigen	NC	++	+++	Yes	6.5–8.5	+++	+	+++
	Lectins‐glycoprotein	NC	+	++	Yes	6–7.4	+++	+	+++

* Qualitative table highlighting the relative comparisons of each strategy to provide information on the considerations.

Abbreviation as follows: C = covalent bonding; NC = non‐covalent bonding.

## 8 Protein Particles in Nanomedicine

Thus far, the evolution of PNPs as drug delivery vehicles, the methods to produce them and examples of PNPs produced by that method were discussed. Understanding the fabrication methods is important in the field of nanotechnology because it largely dictates the nanoparticle properties which are desired according to the application. The application of nanoparticles in the field of medicine, or more specifically, the use of nanotechnology to diagnose, prevent, and treat diseases, is referred to as nanomedicine. Nanomedicine largely seeks to improve patient outcomes by addressing limitations associated with current drugs formulations, especially poor bioavailability. Other potential advantages exist that are responsible for the traction it has received; nanoparticles can alter solubility, stability, and bioavailability of various types of therapeutics including hydrophobic or hydrophilic small molecules, peptides, and nucleic acids.^[^ ^2^, ^314^, ^315^, ^316^, ^317^, ^318^ ^]^ Additionally, blood circulation half‐life and tissue targeting can be augmented by decorating nanoparticles with stealth moieties or targeting ligands.^[^ ^319^ ^]^ The release of encapsulated drugs can be precisely tuned to maintain the drug concentration within its therapeutic window over a prolonged period of time.^[^ ^320^ ^]^ Ultimately, these nanoparticle‐mediated features enable an increase in the amount of drug in the target cells while minimizing systemic toxicity. The translation of nanoparticles within nanomedicine was spearheaded by Doxil (doxorubicin liposome), the first nanomedicine to receive FDA approval in treating AIDS‐associated Kaposi's sarcoma.^[^ ^321^ ^]^ Other milestones include the delivery of synergistic ratios of two drugs (VYXEOS) in 2017 and the first FDA‐approved RNAi therapy based on lipid based nanoparticles (Patisiran/ONPATTRO) in 2018.^[^ ^322^ ^]^

### 8.1 Bottlenecks and Opportunities for Nanoparticle Delivery Strategies

Despite the impressive progress made in the field of nanomedicine, with clear evidence of early successes,^[^ ^322^ ^]^ broad clinical translation of novel nanoparticle platforms is still limited due to a series of bottlenecks.^[^ ^323^ ^]^ Nanoparticles suffer from rapid clearance from circulation, accumulation in the liver, inefficient permeation across the endothelium into target tissues and overall inefficient delivery to target cells.^[^ ^324^ ^]^ A meta‐analysis of 232 data sets revealed that only a median of 0.7% of the administrated nanoparticle dose was able to reach to a solid tumor.^[^ ^325^ ^]^ These data sets included particles with organic and inorganic materials, active and passive targeting strategies, various hydrodynamic diameters, zeta potential, and shape. Surprisingly, the median delivery efficiency showed no improvement over 2005–2015, the 10‐year period of the surveyed literature.^[^ ^325^ ^]^ These findings underscore a pressing need for the design and development of alternate drug delivery platforms that are adequately equipped to address these barriers.

**Figure 9:**
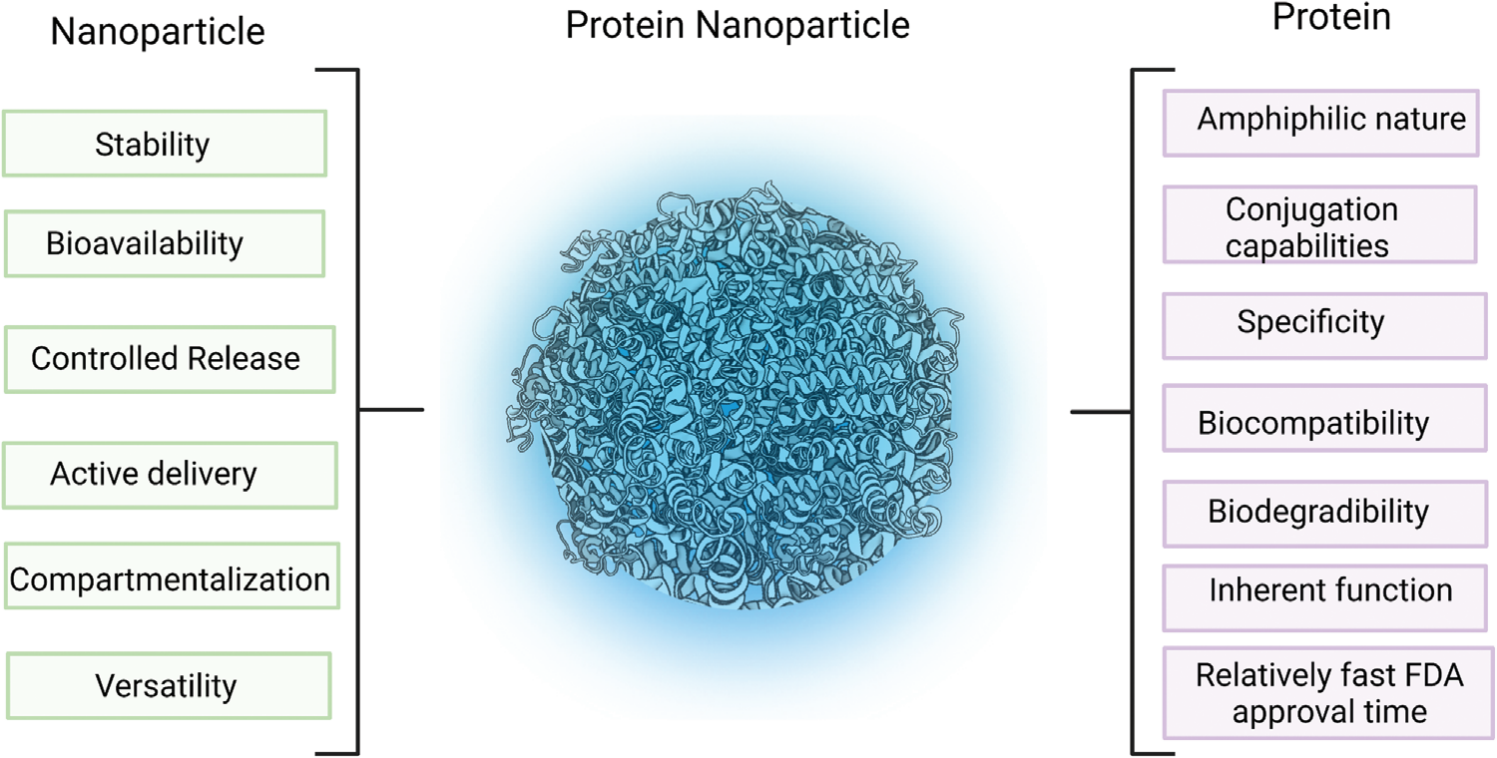
Nanoparticle (NP) delivery systems and protein‐based therapeutics each consist of advantages that are captured within the PNP technology. Created with BioRender.com.

One promising approach is the use of proteins as structural components in nanoparticle‐based drug delivery systems.^[^ ^323^ ^]^ Protein‐based nanomedicine is founded on the premise to explore the endogenous properties of proteins to design carrier systems with improved drug delivery profiles. The advantages of PNPs can be delineated by dissecting the individual components of PNP: nanoparticles and proteins. In the **Figure** **9**, the benefits of nanoparticles and proteins for drug delivery are listed. The intersection between these are the benefits of PNPs in nanomedicine.

Properties of PNPs that make them more attractive than their traditional counterparts include: i) biocompatibility, ii) biodegradability, iii) versatility, iv) chemical conjugation capabilities, v) low immunogenicity, and vi) unique endogenous mechanisms that can be leveraged to address the challenges faced by traditional drug delivery systems. Their primary structure with various chemically reactive side groups allows for effective surface modifications.^[^ ^35^, ^106^ ^]^ Moreover, the amphiphilic nature of proteins enables encapsulation of both, hydrophilic and hydrophobic drug molecules.^[^ ^89^, ^107^ ^]^ Recombinant protein technologies can provide access to a variety of protein building blocks with precisely engineered functions.^[^ ^46^, ^326^, ^327^ ^]^ Consequently, a variety of proteins such as hemoglobin, mucin, transferrin, and HSA have been employed to produce PNPs to leverage their innate function and properties.

### 8.2 Hemoglobin Nanoparticles

The transportation of oxygen and carbon dioxide to and from tissues and organs relies on red blood cells (RBCs) and more specifically, the transport protein hemoglobin (Hb), abundantly found in RBCs.^[^ ^328^ ^]^ Hemoglobin is a tetrameric protein that can reversibly bind to O_2_ by iron atoms coordinated by heme groups.^[^ ^328^ ^]^ Its exceptional oxygen binding capabilities make it a crucial protein for the development of semi‐artificial red blood cells with extended half‐lives.^[^ ^329^ ^]^ To improve upon the short circulation times and low bioavailability of hemoglobin PNPs, more advanced Hb‐based oxygen carriers (HBOC) have been developed.^[^ ^329^ ^]^ To protect hemoglobin from becoming oxidized into methemoglobin, its inactive counterpart, Hosaka et al. designed PNPs with a hemoglobin core and a HSA shell that was decorated with Pt nanoparticles.^[^ ^330^ ^]^ Due to its reducing properties, the HSA‐Pt shell was capable of protecting the hemoglobin from oxidation. Preclinical studies into the safety of PNPs decorated with Pt clusters found longer half‐lives, no negative effects on vital organs, and serum biochemical markers that indicated no abnormalities when compared to the control group.^[^ ^331^, ^332^, ^333^ ^]^

### 8.3 Mucin Nanoparticles

Mucins represent another type of protein that has been used as the carrier material with inherent function. Mucins are amphiphilic, high‐molecular weight glycosylated protein found in epithelial cells and tissues and are rich in threonine and/or serine amino acid residues.^[^ ^334^ ^]^ Their coatings in cavities and tracts creates a protective barrier due to their adhesive properties that can be traced back to repeated structures of hydrophilic and hydrophobic domains.^[^ ^335^ ^]^ Photosensitizer‐loaded gold nanoparticles were embedded in mucin to improve biocompatibility and transport across mucosal barriers to ultimately enhance the long‐term stability of the photosensitizer.^[^ ^336^ ^]^ Thasneem et al. produced PLGA nanoparticles with mucin conjugated to mitigate initiation of immune responses and to improve circulation.^[^ ^335^ ^]^ Mucin has also been used as a stimuli‐responsive nanoparticle system for site‐specific delivery and to minimize off target effects. Kimna et al. produced mucin‐based DNA crosslinked nanoparticles capable of effectively delivering cargo only when a particular DNA sequence was cleaved and release was triggered.^[^ ^337^ ^]^ The mucin PNPs were produced by leveraging their previous finding that that mucins condensate in the presence of glycerol, thus reducing the size by an order of magnitude.^[^ ^338^ ^]^ The resulting mucin nanoparticles could then be stabilized through cationic crosslinkers. Stimuli responsive mucin nanoparticles were designed by covalently conjugating the protein with a crosslinker DNA that contained a partially self‐complementary region.^[^ ^337^ ^]^ This region was intended to form temporary crosslinks that were displaced in the presence of a fully complementary DNA sequence. The authors demonstrated intracellular release of interest through the conformational change which was initiated DNA displacement.

### 8.4 Transferrin Nanoparticles

Transferrin is another widely studied protein for nanomedicine applications. It is responsible for the transportation of iron throughout the body.^[^ ^339^ ^]^ When iron binds to the transferrin, it undergoes a conformational change that allows it to specifically recognize transferrin receptors highly expressed in immature erythroid cells, placental tissue, and rapidly dividing cells.^[^ ^340^ ^]^ It then becomes internalized through the transferrin‐mediated transcytosis pathway and the bound iron is released due to a change in endosomal pH.^[^ ^341^ ^]^ This transcytosis pathway has been of interest to facilitate transport of therapeutics across biological barriers like the blood brain barrier and to achieve targeting to cells, especially malignant cells for cancer applications.^[^ ^342^, ^343^, ^344^ ^]^ Functionalized nanoparticles with transferrin or nanoparticles constructed completely from transferrin has been conducted.^[^ ^150^, ^342^, ^345^, ^346^ ^]^ While there are promising reports of transferrin being used as a carrier and targeting material, there are concerns with the preservation of targeting capabilities once in vivo. Specifically, it has been reported that the formation of biomolecular coronas around transferrin‐functionalized nanoparticles can render the targeting properties inactive.^[^ ^347^ ^]^

### 8.5 Albumin Nanoparticles

The arguably most important protein to be used as a protein carrier with intrinsic function is albumin. HSA, the most abundant plasma protein, is responsible for the circulation of hydrophobic nutrients and hormones throughout the body and represents the gold standard in protein for nanomedicine technology.^[^ ^348^ ^]^ Its popularity as a carrier can be attributed to several characteristics. The first is stability; HSA is stable at a pH range between 4 and 9 and at temperatures as high as 60 °C for up to 10 hours.^[^ ^106^ ^]^ Second, albumin has a few transport benefits; i) it can bind to cell‐surface receptors such as glycoprotein 60 and mediate endothelium transcytosis,^[^ ^349^ ^]^ ii) it may cross mucosal barriers through receptor mediated transcytosis by engaging with receptors such as the neonatal Fc receptor,^[^ ^350^ ^]^ and iii) transportation and accumulation to tumors and inflamed tissues are preferential.^[^ ^108^, ^351^ ^]^ As an example of leveraging targeting, albumin nanoparticles targeted glioblastoma tumors through the overexpression of albumin‐binding proteins on the tumor vessel endothelium: secreted protein acidic and rich in cysteine (SPARC) and glycoprotein 60.^[^ ^94^ ^]^ These albumin PNPs, produced via EHD jetting, were encapsulated with the siRNA against Signal Transducer and Activation of Transcription 3 (STAT3) factor and contained the tumor penetrating peptide, iRGD for glioblastoma therapy.^[^ ^94^ ^]^

**Figure 10:**
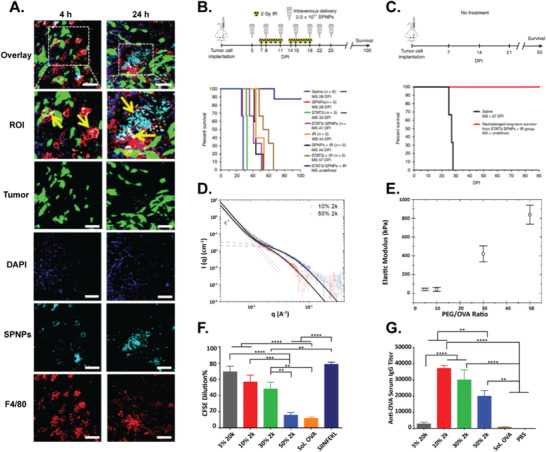
Different characterization and applications of EHD‐fabricated PNPs. A) Kaplan–Meier survival curve. Long‐term survival timepoint was achieved for mice treated with STAT3i and IR. B) Kaplan–Meier survival curve. All the rechallenged survivor mice reached a second long‐term survival timepoint. C) Localization of tail vein administered STAT3i PNPs (cyan) in the brains after 4 and 24 h. (scale bar: 50 µm) Macrophages are shown in red and tumor cells are shown in green. Reproduced with permission.^[^ ^94^ ^]^ Copyright 2020, Springer Nature. D) Small angle neutron scattering of ovalbumin PNPs with 10% and 50% PEG/OVA ratio. E) Elastic modulus of ovalbumin PNPs with varying PEG/OVA ratios. F) CD8+ T cells proliferation percentage after co‐culture with ovalbumin PNPs treated BMDCs. G) Serum anti‐OVA IgG titer on day 20 after subcutaneous injection of OVA PNPs. Reproduced with permission.^[^ ^92^ ^]^ Copyright 2020, Wiley‐VCH.

These PNPs were found to localize in an aggressive GL26 syngeneic mouse glioma model after intravenous administration. Confocal images in **Figure** **10**A show that a significant number of PNPs were able to cross the blood‐brain barrier and localized in the tumor microenvironment.^[^ ^94^ ^]^ Combination of systemic delivery of STAT3i PNPs with the current standard of care, focused radiotherapy, resulted in tumor regression and reaching long‐term survival in 87.5% of GBM‐bearing mice (Figure 10B).^[^ ^94^ ^]^ Remarkably, when the survivors from this treatment group were rechallenged with GBM with no further treatment, they survived to a second long‐term survival point, indicating anti‐GBM immunological memory development (Figure 10C).^[^ ^94^ ^]^

Third, albumin is readily available. Albumin can be derived from other sources like egg whites (ovalbumin, OVA) or bovine (bovine serum albumin) and has been investigated in nanomedicine applications. In another study by Lahann and coworkers, ovalbumin nanoparticles were produced via EHD jetting and stabilized with a PEG‐NHS crosslinker for cancer immunotherapy.^[^ ^92^ ^]^ The physicochemical properties of PNPs were finely tuned by controlling the macromer to protein ratio (PEG/OVA) to improve antigen delivery and immunological responses. Small angle neutron scattering measurements on PNPs with 10% and 50% PEG/OVA demonstrated that *ξ*, the average spacing, decreased by nearly twofold with increasing the PEG/OVA ratio (Figure 10D). As measured by AFM the elastic moduli of PNPs increased as the PEG/OVA ratio was increased (Figure 10E). The physicochemical properties of these PNPs directly influenced their cellular and humoral immune responses: OVA PNPs with lower PEG/OVA ratios led to improved CD8+ T cell activation in vitro and enhanced draining lymph node delivery, antibody production, and anti‐tumor efficacy in vivo. Specifically, the OVA particles with 10% PEG/OVA ratio resulted in 4.4‐fold higher CD8+ T cell proliferation rate compared to particles with 50% PEG/OVA ratio (Figure 10F). Moreover, the anti‐OVA serum IgG titers induced after prime immunization with 10% PEG/OVA PNPs was 1.9‐fold and 49.2‐fold higher compared to 50% PEG/OVA PNPs and solute OVA, respectively (Figure 10G). Fourth, since albumin can transport endogenous hydrophobic material, it has been exploited to carry synthetic hydrophobic materials. This is highlighted with commercialization of Abraxane, the nanoparticle albumin paclitaxel technology, which encapsulates the hydrophobic paclitaxel within HSA domains. Since Abraxane's success, others have used albumin to deliver alternative hydrophobic drugs like docetaxel. In one study, docetaxel‐loaded HSA PNPs were developed and decorated with folic acid by covalent conjugation to interfacial amine groups to promote binding to overexpressed folate receptors in tumor cells.^[^ ^20^ ^]^

### 8.6 Alternative Protein Nanoparticles

Other proteins have been used in nanomedicines, but not as both the medicine and structural component. For example, insulin nanoparticles have been produced, but they are susceptible and vulnerable to becoming inactive and have largely been investigated as a cargo since protection is necessary.^[^ ^352^, ^353^ ^]^ Similarly, other proteins have been used as the structural component rather than eliciting any specific effect inherent to that protein.^[^ ^354^, ^355^ ^]^

## 9 Outlook

### 9.1 Protein Nanoparticle Design Considerations

Proteins as natural polymers can be heterogenous mixtures of different molecular weights which can lead to differences in product characteristics and batch‐to‐batch variabilities.^[^ ^356^, ^357^ ^]^ In addition, due to proteins natural origin, they can be contaminated with pathogens from other species.^[^ ^358^, ^359^ ^]^ Even with the advent of producing recombinant proteins using genetic engineering techniques, they can still be contaminated with low levels of bacterial products, like witnessed for proteins produced in Escherichia Coli.^[^ ^46^, ^326^, ^327^, ^360^ ^]^ These contaminations can include lipopolysaccharide (also referred to as endotoxin), bacterial DNA, and outer wall proteins which can induce immunogenic responses.^[^ ^360^, ^361^ ^]^ Therefore, the removal of bacterial endotoxin from the final recombinant protein or developing methods for the production of endotoxin‐free protein is necessary to ensure the safety of the final product.^[^ ^362^ ^]^

The immunogenicity profiles of PNPs are one of the major considerations for both their safety and efficacy when used as drug delivery nanocarriers.^[^ ^363^ ^]^ The most common effect of immunogenic protein‐based therapeutics is the development of a high affinity anti‐therapeutic antibody response which can reduce or eliminate their therapeutic effects.^[^ ^364^, ^365^, ^366^ ^]^ Clinical studies have shown that protein‐based therapeutics derived from endogenous human proteins or foreign proteins with little human homology are capable of stimulating undesirable immune responses.^[^ ^363^, ^364^ ^]^ Although recombinant DNA technologies are utilized to engineer recombinant proteins to reduce their immunogenicity,^[^ ^367^, ^368^ ^]^ it should be noted that production of proteins by genetic engineering methods results in an increase in their production cost.^[^ ^356^ ^]^ The differences in the 3D structures of protein‐based therapeutics and natural proteins can also trigger B cells to produce antibodies against therapeutic proteins.^[^ ^366^ ^]^ Another structural component of PNPs which may also contribute to the nanoparticles immunogenicity is the chemical compound or macromer used to stabilize the PNPs. As an example, polyethylene glycol (PEG)‐based bifunctional macromers have been utilized to crosslink PNPs^[^ ^91^, ^92^, ^94^ ^]^ or surface conjugation of PEG, known as PEGylation, has led to bringing more than ten protein‐based therapeutics into market.^[^ ^369^ ^]^ In this approach, PEG is used as a “non‐fouling” material to resists non‐specific protein adsorption on nanotherapeutics and prolong circulation half‐life.^[^ ^369^, ^370^ ^]^ In addition, PEGylation has been used to shield antigenic epitopes of nanotherapeutics from immune system recognition and thus mitigate immunogenicity.^[^ ^369^ ^]^ Despite this success, there is a growing body of literature that shows the presence of anti‐PEG antibodies, as an innate immune response, which has been further correlated with the loss of therapeutic efficacy and increase in adverse effects after repeated administrations.^[^ ^369^, ^371^, ^372^ ^]^ Since immune responses may lead to neutralizing the activity of highly effective protein‐based therapeutics and inducing hypersensitivity responses including anaphylaxis,^[^ ^363^ ^]^ the choice of protein, PNP fabrication method, and the preservation of the protein's native structure during PNP fabrication process are among the key factors influencing the PNP immunogenicity and ultimately their clinical efficacy.

Another consideration when designing PNPs for targeted drug delivery is their unique cell‐particle interactions in the body due to the innate properties of proteins as natural biomolecules. It was recently shown that PNPs with agglutinated protein accumulate in pulmonary marginated neutrophiles during acute inflammation.^[^ ^149^ ^]^ This intrinsic tropism for inflamed lungs highlights that protein‐based nanotherapeutics could be a potential platform for treatment and diagnosis of inflammatory disorders such as acute respiratory distress syndrome or other disorders in which neutrophils are key players. However, these findings demonstrate that many protein‐based nanoparticles may accumulate in inflamed lungs, even when they were designed and intended to target another organ, as an off‐target effect.^[^ ^149^ ^]^

Achieving a proper drug release profile is one of the major design criteria for engineered PNP as drug carriers as it is one of the hallmark benefits for implementing nanoparticle delivery strategies. However, rapid release has been reported when using proteins and PNPs.^[^ ^356^ ^]^ To mitigate this, various strategies for stabilizing PNPs and controlling the mesh size of crosslinked PNPs have been investigated to facilitate slower diffusion rates in optimizing the cargo release profile.^[^ ^91^, ^92^ ^]^

Another notable challenge with PNP is their limited oral delivery potential. The parenteral administration is currently the most commonly used administration route for PNPs.^[^ ^373^, ^374^ ^]^ This is primarily due to i) low pH of the stomach leading to hydrolysis of PNPs, ii) digestion of PNPs by stomach enzyme (pepsin), and small intestine degradative enzymes (pancreatin, trypsin, amylase, maltase, and lipase), iii) challenges with overcoming mucus layer of intestine and bypassing the first‐pass effect to reach the circulation system. Collectively, extreme pH conditions, catalytic enzymes and the mucosal layer hinder the oral delivery of PNP.^[^ ^373^ ^]^ The inability to rely on oral delivery as a method of administration may result in poor patient compliance, making it a significant drawback of this delivery strategy.

Despite the disadvantages associated with PNP drug delivery, they still offer advantages that warrant their exploration in the field. These advantages range from versatility, biodegradability, design space, and relatively low immunogenicity. What makes PNP delivery exceptional compared to its competitors, is the breadth of design capabilities available at scientists’ fingertips: the selection of protein, stabilization strategy, fabrication method, post‐fabrication processing, and conjugation techniques (**Table** **9**). The PNP field is unique in that the development is contributed by the growth of protein therapeutics whether that be engineering techniques or the discovery and characterization of proteins structures.

**Table 9:** A qualitative analysis of design considerations for various PNP fabrication methods

Technique	Versatility*	Modularity**	Loading efficiency	Mono‐dispersity of PNPs	Protein structure retainment	Throughput	Economical instrumentation	Process simplicity
Emulsification	++	+	+	+	++	++	+++	+++
Nab‐technology	+	+	++	++	+	+++	+	++
Desolvation	+	++	++	+++	+	+++	+++	+++
EHD	+++	++	+++	++	+++	++	+++	+++
Self‐assembly	+	+++	+	+++	+++	+	+	+

* Versatility: Applicability of different protein‐solvent systems;

** Modularity: Control over size, shape, topology, roughness, or other physicochemical properties

### 9.2 Clinical Projections

**Figure 11:**
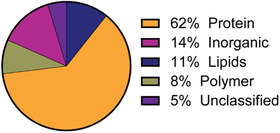
Clinical trials from 2001 to 2021 with the search term, “nanoparticles” on clinicaltrails.gov.

To further understand how PNPs fare against other NP systems in recent years, a brief clinical trial search was conducted. The increased interest in PNPs can be deduced from a review of recent nanoparticle formulations that were evaluated in clinical trials. Based on clinicaltraisl.gov from 2001 to 2021, it is evident that there have been more protein‐based particles in the development pipeline than any other materials class (**Figure** **11**). Categorizing the clinical trials based on the nanoparticles composition (lipids, protein, polymer, inorganic, and unclassified) demonstrates protein‐based nanoparticle drug delivery systems gained a lot of attention comprising 62% of the total 234 clinical trials. Although the clinical success of protein nanoparticles is dominated by Abraxane, the field of protein nanoparticles is yet emerging and there is the potential to expand the protein nanoparticles beyond Abraxane. Further clinical translation of PNPs critically hinges upon the availability of suitable manufacturing processes.

## 10 Conclusions

Protein‐based nanoparticles have long been used for a wide range of industrial and food applications. Their favorable property profiles, including on‐demand degradability, a broad range of functional groups for subsequent modification, and their natural ability to interact with biopharmaceutical drugs have placed them at the forefront of nanomedicine in recent years. While a wide range of methods exists to formulate PNPs with well‐defined properties, including desolvation, emulsification, and Nab technologies, those methods that do not require the use of organic solvents and limit the need of chemical crosslinkers will likely gain interest in the future. One direction of research will likely focus on self‐assembly processes that leverage major progress in protein engineering and computational biology to develop tailor‐made proteins as building blocks of future PNPs. Another direction will likely use atomization methods to create more complex PNPs, including multicompartmental nanoparticles. The latter will be ideal carrier systems for combination drug delivery. The fact that highly dissimilar drugs can be compartmentalized within hemispheres of the same particle and released with tunable release kinetics has the potential to address major challenges in targeted drug delivery. Based on the astonishing progress in this field in recent years, the concept of the “golden bullet” in targeted delivery may finally be within reach.

## Conflict of Interest

The authors declare no conflict of interest.
